# Novel genetic variants identification and immune profiling in ataxia telangiectasia patients

**DOI:** 10.1186/s12967-026-07871-2

**Published:** 2026-02-19

**Authors:** Rim Jenni, Hedia Klaa, Asma Chikhaoui, Khouloud Zayoud, Emmanuelle Cochet, Ichraf Kraoua, Lydie Burglen, Houda Yacoub-Youssef

**Affiliations:** 1 Laboratory of Biomedical Genomics and Oncogenetics (LR16IPT05), Institut Pasteur de Tunis, University Tunis El Manar, Tunis, 1002 Tunisia; 2 LR18SP04 and Department of Child and Adolescent Neurology, National Institute Mongi Ben Hmida of Neurology, Tunis, 1007 Tunisia; 3 Unit of Pediatric Neurogenetics, Department of Genetics, Trousseau Hospital, APHP.Sorbonne University, Paris, 75012 France; 4 Reference Center for Malformations and Congenital Diseases of the Cerebellum and Unit of Pediatric Neurogenetics, Department of Genetics, Trousseau Hospital, APHP. Sorbonne University, Paris, 75012 France

**Keywords:** Ataxia telangiectasia, ATM, Bioinformatics, Immunophenotyping, Gene expression analysis

## Abstract

**Background:**

Ataxia telangiectasia (AT) is an autosomal recessive neurodegenerative disease. While heterozygous relatives of AT patients are known to be clinically healthy, a predisposition to various pathologies has been reported. Our aim was firstly, to further characterize the clinical features and broaden the spectrum of genetic pathogenic variants in AT patients. Secondly, we aimed to study the immune profiles of AT patients and their relatives to identify similarities or common biomarkers.

**Methods:**

A Target Gene Sequencing for six patients suspected with AT was performed. Computational analysis was conducted to assess the pathogenicity of novel variants. The distribution of immune cells was assessed by flow cytometry in patients with AT, AT-like disorder, Friedreich ataxia, and in AT relatives. The expression pattern of candidate genes was evaluated by RT-qPCR.

**Results:**

We identified and predicted the pathogenicity of novel variants in the *ATM* gene. Computational analysis suggested that the novel identified missense mutation could affect ATP binding pattern and ATM protein flexibility, while Alu element insertion could probably induces a premature stop codon. Furthermore, our results confirm the pathogenic effect of identified splicing mutations on the ATM transcript. Moreover, we noticed a high percentage of LTCD4 + and LTCD8 + senescent subsets in AT patients and a relative increase of the of intermediate and non-classical monocytes accompanied with a decrease of classical monocytes specifically in AT patients with truncated biallelic mutations which was intriguingly similar to the immune profile of AT parents. In addition, a difference of immune pattern was observed between AT patients with biallelic truncated mutations compared to those with at least one non-truncated mutation, with a variability intragroup. Gene expression analysis identified *FOXO3*, *IL33* and *METTL3* as putative genes that may yield clues into AT pathogenesis.

**Conclusion:**

Taken together, our study expands the mutational spectrum of AT disease worldwide and further characterize the immune profile of AT patients uncovering a possible difference in some immune cellular subsets related to *ATM* mutation type and delineate putative immune abnormalities related to *ATM* heterozygosity among AT parents. Furthermore, dysregulation in *FOXO3*, *IL33* and *METTL3* expression could be related to disease severity.

**Supplementary Information:**

The online version contains supplementary material available at 10.1186/s12967-026-07871-2.

## Background

Ataxia telangiectasia (AT) is a rare and severe multisystemic neurodegenerative disease. It is the most common autosomal recessive cerebellar ataxia (ARCA) after Friedreich ataxia. AT is characterized mainly by cerebellar ataxia, immunodeficiency, oculocutaneous telangiectasia, cancer predisposition and radiosensitivity [1].

AT is due to a biallelic mutation in the *ATM* gene coding for the ATM kinase protein. This protein regulates cellular homeostasis initiating a wide spectrum of signal transduction pathways, through activating an array of proteins involved in DNA repair, RNA metabolism, autophagy, apoptosis, mitochondrial function and oxidative stress [2]. Indeed, ATM is a key regulator of DNA Damage Response (DDR). This function is particularly crucial for immune cells, as ATM plays a critical role in the repair of DNA damage induced during V(D)J recombination and class switch recombination (CSR), which is an important process for immune repertoire diversity [3, 4].

Malignant diseases and chronic pulmonary pathologies due to recurrent sinopulmonary infections are the two major leading cause of premature death among AT patients, both are presumably related to immune dysfunctions [1]. Indeed, the high sensitivity to ionizing radiation and the excessive toxicities to chemotherapy observed in AT patients, add a level of complexity posing an immense challenge for the clinical management of cancer [5]. It was reported that TTI1-mediated ATM activation is involved in irradiation-induced DNA damage repair underlying tumor radioresistance and that inhibition of ATM signaling could trigger radiosensitivity [6]. Nonetheless, AT patients exhibit hypersensitivity to irradiation associated with ATM deficiency inducing cytotoxic effects upon exposure, limiting therapeutic options for patients with cancer [7]. Furthermore, ataxia, which is the most debilitating feature of AT disease, due to progressive neurodegeneration of the cerebellum has been tightly linked to microglia activation and neuroinflammation which precede neuronal cell death [8]. These highlight the importance of the immune response in AT pathology underscoring the need for better characterization of the immune profile of AT patients and understanding its involvement in the pathology.Immunological deficiencies in humoral and cellular components have been reported among AT patients with different degree of severity varying from a mild alteration of the humoral immunity to a severe combined immunodeficiency [9, 10]. Immunoglobulin deficiency, notably in IgA is a well-studied immune abnormality in AT patients, although, an hyper IgM phenotype has been associated with a more severe clinical phenotype with decreased life expectancy [11]. B- and T-cell lymphopenia has also been widely documented [12]. Regarding the T-cell population, AT is characterized by a reduced rate of naïve LT (CD45RA^+^) CD4 and CD8 T cells, while memory T cells (CD45RO^+^) remain within the normal range [12, 13]. In addition to functional abnormalities in T lymphocytes, alterations in the NK cell compartment have also been reported with conflicting data. While prior studies described changes in the frequency of NK cells, others rather pinpoint to functional alterations alongside a preserved cell frequency [12, 14, 15]. The immune system of AT patients has been characterized as congenitally aged which may underly immunodeficiency observed in patients [16, 17]. Indeed, immunosenescence and inflammaging are key hallmarks of immune aging. Inflammaging is a chronic low-grade inflammation that contributes to immunosenescence, which consists of a decline in the function of cells involved in innate and adaptative immunity. Inflammaging and immunosenescence, are interconnected creating a vicious cycle accelerating the aging process and contributing to age-related diseases. Furthermore, cellular stress and accumulation of DNA damages could define cell fate, among which could trigger cellular senescence. The persistent production of senescence associated secretory factors (SASP) by senescent cells further exacerbates the inflammatory state and promotes inflammaging [18, 19]. Chronic inflammation and premature aging are important components in AT related to different disease phenotype, highlighting their potential contribution to disease pathogenesis [20, 21]. They have been also associated to the neurodegenerative process in AT patients [22]. Moreover, gene expression profiling revealed dysregulation of genes involved in immunity and inflammation pinpointing to a relationship between immune defects in AT patients [23]. A better understanding of alterations in immune cell sub-populations could gain insights into disease pathogenesis.

At genetic standpoint, different mutations in the *ATM* gene have been identified defining classical and variant AT patients. While truncating mutations result in the total loss of ATM protein and/or of its kinase activity leading to a severe classical phenotype, leaky splice site and missense mutations result usually in a residual kinase activity leading to a variant AT phenotype characterized by a milder phenotype with late AT onset and slower progression rate [24, 25]. Although previous research studies attempted to explore genotype-phenotype correlation in the context of AT disease, few findings regarding the immune profile in these patients have been reported, with heterogeneous results [26–29]. Given the importance of the immune component in AT disease, understanding the correlation between *ATM* mutation and immune pattern could help identify patients with specific causative variants that could be associated with a more severe immunological phenotype requiring a close monitoring and a personalized follow-up for a timely management avoiding further complications.

Furthermore, although parents of AT patients are known to be clinically healthy carriers, epidemiological studies have shown an increased health risk associated with ATM heterozygosity, characterized by cancer predisposition, high risk of ischemic heart diseases and diabetes, high infections episodes, suggesting a similarity to patients with AT [30]. Susceptibility to infections and the age-related diseases raises the question of whether carriers of *ATM* mutations, notably parents of patients could have immune alterations similar to those seen in AT patients as they share certain cellular deficiencies with AT patients, such as chromosomal abnormalities and radiosensitivity [31, 32]. Moreover, only one report has examined the immune profile of AT patients’ parents, revealing certain similarities in immune cells that could be associated with increased DNA damages [33].

AT disease shares clinical features and molecular mechanisms with other forms of ARCA, notably with Ataxia telangiectasia-like disorder 1 (ATLD1) and Friedreich ataxia (FA), nevertheless, the immune cells’ profile of patients affected with these diseases remains poorly investigated [34, 35].

Despite that extensive research have been conducted to better understand AT disease, no curative therapy is currently available which may be linked to the lack of molecular insights into disease pathogenesis and biomarkers that permit to assess clinical severity, track progression and monitor response to therapy. Peripheral blood has been emerged as surrogate tissue for reliable and easily accessible biomarkers identification in neurodegnerative diseases [36–38]. Although studies based on gene expression profiling of peripheral blood mononuclear cells (PBMCs) from patients with AT are scarce, they have reported an association of the transcriptional pattern with the phenotypic variability, highlighting their relevance for disease classification and the identification of putative biomarkers [23, 39, 40]. In this study, we aimed firstly, to analyze the clinical features and the genetic landscape of AT Tunisian patients. Secondly, we sought to characterize the immune cell profiles in AT patients, their relatives as well as in other forms of ARCA (ATLD1 and FRDA), mainly in the AT group and to attempt to further explore the possible relationship between genetic defect and immune profile in AT patients. Finally, we aimed to conduct a gene expression analysis to investigate transcriptional changes in immune cells that could be related to clinical severity in AT disease.

## Materials and methods

### Patients

The study was carried out in accordance with the Helsinki principles and was approved by the Biomedical Ethics Committee of Institute Pasteur of Tunis (IPT). The ethical approval number is (2022/4/I/LR16IPT/V2).

Patients were recruited from the Department of Pediatric Neurology of the National Institute of Neurology Mongi Ben Hamida. The clinical diagnosis of AT, ATLD1 and FRDA diseases was established by pediatric neurologists based on different examinations that were initiated upon suspicion of ataxia; including clinical neurological assessment, brain Magnetic Resonance Imaging (MRI), Electromyography (EMG), and blood tests. Clinical diagnosis of AT was made based on the core clinical hallmarks of the disease, as described previously by Micol et al. AT diagnosis is made when at least three of the following criteria are met: ataxia, oculocutaneous telangiectasia, recurrent sinopulmonary infections, IgA deficiency, high serum level of a-fetoprotein (AFP), or AT karyotype abnormalities [41].

ATLD1 patients present AT-like features with milder phenotype and slower progression rate. Indeed, the presence of an early onset cerebellar ataxia with absence of telangiectasia and normal AFP level are more suggestive of ATLD1 disease. The clinical diagnosis of early onset FRDA is based on the neurological manifestations, notably the presence of ataxia, decreased vibration sensation and reflex loss in the lower extremities, usually accompanied with non-neurological symptoms notably cardiomyopathy, scoliosis and diabetes mellitus. A relatively later onset of ataxia with milder phenotype and slower progression are usually diagnostic criteria suggestive of ATLD1 and FRDA rather than AT disease. After obtaining written informed consents from patients’ legal guardians, parents and donors, blood samples were collected. Based on a questionnaire we conducted, participants with an active infection at the time of diagnosis or immediately prior to diagnosis, or those taking medication that could impact their immune profile were excluded. Samples were processed according to the laboratory protocols and conserved under standard biobank conditions. Clinical, epidemiological and genealogical data were compiled from existing patients’ medical records. Patients with missing or incomplete key clinical data were omitted from the study. All samples were pseudonymized prior to analysis. Genetic pedigrees were designed using the QuickPed online tool.

### Mutational screening

DNA was extracted from peripheral blood of six patients with a clinical diagnosis of AT disease, using the FlexiGen DNA Kit (Qiagen) according to the manufacturer’s protocol. DNA quality and concentrations were assessed by a Nanodrop Spectrophotometer DeNovix DS-11 (Thermo Fisher Scientific). Genetic investigation was performed at the Reference Center for Malformations and Congenital Diseases of the Cerebellum and Laboratory of Molecular Neurogenetics, Department of Genetics, Armand-Trousseau Hospital in Paris, France. A targeted gene panel sequencing was conducted using NGS sequencing of a cerebellar-movements disorders panel CEREBMD_V2 kit targeting 247 genes involved in cerebellar disorders, including ATM gene. Samples were sequenced on a NextSeq 500 (Illumina) aiming at 30X for at least 98% of target regions, with an average depth of at least 500x. Variant calling was performed with a detection threshold set at 30x. Reads were aligned/mapped to the Genome Reference Consortium Human Build 38 (GRCh38). Custom-made bioinformatic pipeline G-route and Leaves software (pipeline version CCIP 11.8.24) were used for SNP and CNV analyses. The variants were classified according to the American College of Medical Genetics and Genomics and Association for Molecular Pathology (ACMG/AMP) guidelines.

All identified putative pathogenic variants were confirmed by Sanger sequencing performed by the ABI Prism 3500 sequencer (Applied Biosystems) using the BigDye Terminator Cycle Sequencing Reaction Kit v3.1. A set of primers was designed using the Primer3 Plus software and is presented in Table 1. The PCR was carried out in a Qsp of 25ul, using Master mix, 50ng of gDNA and 10 μm of each forward and reverse primers. RT-PCR followed by sanger sequencing and quantitative real-time PCR (qPCR) were performed after RNA extraction from PBMC to assess the effect of identified splice site mutations on transcript sequence and to quantify ATM mRNA expression level, respectively. QPCR was carried out using ATM-F: GCACGAAGTGCCTCCAATTC and ATM-R: ACATTCTGGCACGCTTTGG. QPCR was performed in duplicate for each sample and results were expressed as fold change. Fold change was calculated after first normalization to the reference housekeeping gene *RPLP0* and relative to healthy controls.

**Table 1:** PCR primer sequences

Exon/Intron	Primer Sequences (5’-3’)	Tm (°C)	Amplicon size (pb)
Exon 14	F: ACAGTACACCCTCCACCCT R: TGCAGATGACGAGTTGATG	56.8 56.7	678
Exon 26	F: GGTGCTACTGAACAAGGTCC R: TCAGAGGGAGACAACACGA	57.2 57.8	685
Intron 38–39	F: AGGACTCTTCAGCCATGT R: ACAGTTCTAACTCAGTCAGAGG	52.8 52.9	878
Intron 48–49	F: CCAACTTCCTTGTACCTCAG R: CACTCCACCCTAGAGACTATACA	54.9 55.2	640
Exon 56	F: GCACAGATGCTCAGATTGG R: ACTTCACCCAACCAAATGG	57.8 58.2	291
Exon 59	F: CCAGACACCCAGTCATTCTG R: ACTCAGTACCCCAGGCAGA	59.1 58.2	868
ADNc 38–39	F: GACACTTCTCGCAAACGAG R: GTTGTACTCTGGCTTCCTTCT	57.1 58.9	331
ADNc 48–49	F: GCCAGAACTTTCAAGAACACTC R: TCCCTAAGGAGACCTACTTCCTC	58.1 59.3	503

### Computational analysis

#### In silico prediction of variants pathogenicity

The frequency of identified variants was assessed using Genome Aggregation Database (gnomAD) browser (v4.0.0) (https://gnomad.broadinstitute.org/) and Exome Aggregation Consortium (ExAC) (https://exac.broadinstitute.org/). The pathogenicity of variants was ascertained through different in silico prediction tools, including mutation Taster (https://www.mutationtaster.org/), ClinVar (https://www.ncbi.nlm.nih.gov/clinvar/), SIFT (https://sift.bii.a-star.edu.sg/), PolyPhen2 (http://genetics.bwh.harvard.edu/pph2/), Varsome (https://varsome.com/), PhD-SNP (https://snps.biofold.org/phd-snp/phd-snp.html), FATHMM-MKL (https://fathmm.biocompute.org.uk/). Sequence conservation analysis was performed using the ConSurf server (https://consurf.tau.ac.il/consurf_index.php). MDEscPredictor tool (https://nmdprediction.shinyapps.io/nmdescpredictor/) was used to assess the effect of frameshift variant. The impact of mutations on the splicing process was examined through SpliceAI (https://spliceailookup.broadinstitute.org/), Human splicing finder (HSF) (http://www.umd.be/HSF/) and Varseak (https://varseak.bio/). To predict the effect of splicing mutation on ATM protein sequence the Expasy tool (https://web.expasy.org/translate/) was explored. Different computational tools were also employed to dissect the effect of mutations at protein level. In fact, protein structural stability upon amino acid substitution was predicted using I-Mutant2.0 (https://folding.biofold.org/i-mutant/i-mutant2.0.html) and MUpro (https://mupro.proteomics.ics.uci.edu/) web servers based on the free energy change (ΔΔG) value. Furthermore, protein structural and functional changes were also examined through different in silico tools; notably MutPred (http://mutpred.mutdb.org/), HOPE (https://www3.cmbi.umcn.nl/hope/input/) and Missense3D (http://missense3d.bc.ic.ac.uk/).

The ATM transcript (NM_000051.4) was used as a reference sequence.

#### Structural modeling and molecular docking

To evaluate how *ATM* novel missense mutation could affect the function of the protein and its interaction with ATP, a molecular modeling and molecular docking were carried out. The reference ATM protein crystal structure was retreived from Protein Data Bank (PDB: 7SIC, resolution: 2.51 Å). Protein structure refinement was performed using Modeller V10.6 through adding missing residues. SCWRL was employed to add side chain conformations (http://dunbrack.fccc.edu/lab/scwrl/) using a server with Operating system Linux—Ubuntu 32.04.3 TLS and Memory/RAM 32 GB RAM. Energy minimization was carried out using YASARA server. The mutagenesis wizard of PyMol was used and best rotamer was chosen. Autodock tools were explored to prepare the ATM protein (receptor) using the upper dimerc structure prior to docking analysis, through removing water molecules, and adding hydrogens and Kollman charges.

The ligand Adenosine-5′-triphosphate (ATP = C10H16N5O13P3) was extracted from PubChem (https://pubchem.ncbi.nlm.nih.gov/). Ligand preparation and optimization of geometry structure was conducted using Avogadro and Autodock tools.

Molecular docking was performed using native or mutant upper dimeric ATM protein structure with its ligand in the presence of the Mg atom through Autodock vina v1.2.7 tool (https://vina.scripps.edu/downloads/). To identify the site-specific docking, the center of the grid box was defined based on the PDB 7SIC, in which the ATM protein is bound to an ATP analog (AMP-PNP) ligand. The coordinates of this ligand permitted to define the grid with a center of mass position in x, y, z axis as 120.83, 166.20, 93.39 and grid size as 30 × 30 × 30 with 0.375 Å spacing, covering the binding-pocket. ChimeraX and PoseEdit of Protein Plus (https://proteins.plus/) were used to visualize ATM-ATP 3D and 2D interaction patterns, respectively.

#### Structural flexibility analysis

To assess the potential effect of identified missense mutation on structural flexibility, a rapid dynamic simulation of protein structure was performed through CABS-flex 2.0 web server (https://biocomp.chem.uw.edu.pl/CABSflex2/) [42] using default settings with 50 cycles.

### Immune cell profiling using flow cytometry

Peripheral blood was collected from AT patients (*n* = 9, mean age = 15.6), their parents (*n* = 6, mean age = 44,8) and age matched healthy donors referred as ctrl-patients (*n* = 3, mean age = 16.3) and ctrl-parents (*n* = 6, mean age = 40). The healthy donors had no health problems or immune deficiencies, and had not received any recent treatment that would have affected their immune systems, according to the questionnaire we conducted. Blood was also collected from patients with other forms of ARCA, notably siblings with Freidreich ataxia (FRDA) (*n* = 2) and siblings with Ataxia telangiectasia like-disorder 1 (ATLD1) (*n* = 2). In order to evaluate a possible correlation between *ATM* genetic variants and immune cell phenotypes, AT patients were, first, classified according to their ATM mutation type into two groups; patients carrying bi-allelic truncating/null mutations were presented as T/T (*n* = 5), while patients with at least one non-truncating mutations notably with missense or splice site mutation as non-T/T (*n* = 4). The immune profil was compared between the two groups. The PBMC were isolated using gradient Ficoll centrifugation technique. Briefly, the freshly collected whole blood was mixed with a same volume of RPMI and gently layered onto the lymphocyte separation medium. A centrifugation was performed, after which the mononuclear cell layer was transferred into a new tube. PBMCs were then washed twice and re-suspended in RPMI and counted using the trypan blue dye. Multicolor flow cytometry was perfomed for 1 × 10^6^ PBMCs/ml of cells, using different combinaison of antibodies to identify different cellular populations; LTCD4 (CD3-APC [BD, Cat. #555342], CD4-PE-Cy5 [BD, Cat. #555348], CD27-PE [BD, Cat. #555441], CD28-APC-H7 [BD, Cat. #561388]), LTCD8 (CD3-APC [BD, Cat. #555342], CD8-FITC [BD, Cat. #555634], CD27-PE [BD, Cat. #555441], CD28-APC-H7 [BD, Cat. #561388]), NK (CD3-APC [BD, Cat. #555342], CD16-FITC [BD, Cat. #555406], CD56-PE-Cy7 [BD, Cat. #557747], CD57-PE [BD, Cat. #560844]), LB (CD19-FITC [BD, Cat. #555412], CD20-PE-Cy5 [BD, Cat. #555624]) and Monocytes (CD14-APC-Cy7 [BD, Cat. #562698], CD16-FITC [BD, Cat. #555406]). A compensation controls was performed for each fluorochrome in the panel using a single stained controls. Plot acquisition was performed on flow cytometer FACSCanto™ II (BD Biosciences) for at least 10.000 events for each analyzed sample. Data were analyzed with the FlowJo vX.0.7 software. The gating strategy is illustrated in the Supplementary file Fig. S1. Gating was performed by a single operator to minimize technical variation. Due to the limited cohort size, no formal statistical testing was performed, and a difference of ≥ 2-fold in measured percentage values in different patient groups compared to controls was used as an indicator of a notable difference. For the comparaison within AT group between T/T and non-T/T groups, all observed differences were documented, given the small sample size and exploratory nature of the study. All data are presented descriptively as median ± IQR, using Graphpad software 9.0.2.

### Gene expression analysis

Total RNA was extracted from PBMC cells of patients (*n* = 6) and age-matched healthy donors (*n* = 2) using the Trizol method and the miRNeasy mini kit (Qiagen), following the manufactures’ instructions. The concentration and purity of isolated RNA were assessed by Nanodrop spectrophotometer DeNovix DS-11 (Thermo Fisher Scientific, Wilmington, DE, USA).

For gene expression analysis, reverse transcription was carried out using the Superscript II RT Kit (Invitrogen) according to the manufacturers’ protocol. The *METTL3*, *FOXO3*, *FOXM1*, *IL33*, *CHCHD2* (also known as *Mnrr1*), *ATF3*, *ATF4*, *PRDX3*, *HTRA2*, *POLG* genes were investigated by quantitative real-time PCR (qPCR) using the LightCycler 480 SYBR Green I Master Mix (Roche). *RPLP0* was used as a housekeeping gene. Q-PCR was performed on a LightCycler^®^ 480 System (Roche Diagnostics). Genes were selected based on their established involvement in cellular stress-related pathways and mitochondrial function (*PRDX3*, *HTRA2*, *POLG*, *CHCHD2*) as reported in the literature. For cellular stress-related pathways, we targeted transcriptional regulators (FOXO family members; notably FOXO3 and FOXM1), stress-inducible transcription factors (ATF family members; ATF3 and ATF4), an epitranscriptomic modifier (METTL3), and a stress-responsive cytokine (IL33), that are linked to stress responses, DNA damage signaling, and inflammatory response. Despite that this gene panel has not been exhaustively studied in AT disease, prior evidence supports the involvement of impaired DNA damage response, stress response mechanisms and mitochondrial dysfunction in this disease providing a significant rationale for evaluating their expression levels in PBMC from AT patients. Furthermore, prior data based on gene expression profiling using different AT models has demonstrated widespread alterations of the transcriptional patterns which further underpin our focus on stress-related and regulatory genes in this study.

Experiments were performed in duplicate for each candidate gene and for the reference gene. Expression values were first normalized to the expression of the housekeeping gene (RPLP0) to correct for sample-to-sample variation. The normalized values were then referenced to the mean expression of the healthy controls and relative expression was determined as fold-change using the 2 − ΔΔCt method. *RPLP0* has been reported as one of the most reliable endogenous controls for qPCR normalization in PBMC. To better illustrate changes in gene expression, we applied a log2 fold-change threshold of − 1 < log2FC < 1 (values <-1 indicate a downregulation while values > 1 indicate an upregulation). Due to the small cohorte size, results are presented descriptively.

Primers were selected from the PrimerBank database (https://pga.mgh.harvard.edu/primerbank/). The sequences of the primers are listed in Table 2.

Hierarchical clustering analysis was performed based on gene expression profiles using Morpheus (https://software.broadinstitute.org/morpheus). Clustering was based on average linkage test and spearman rank correlation, and data was presented as Heatmap.

**Table 2:** Primer sequences used for qPCR

Gene	Primer Sequences (5’-3’)	Length (pb)	Tm (°C)
ATF3	F: CCTCTGCGCTGGAATCAGTC R: TTCTTTCTCGTCGCCTCTTTTT	20 22	62.6 60.2
ATF4	F: CTCCGGGACAGATTGGATGTT R: GGCTGCTTATTAGTCTCCTGGAC	21 23	61.5 62.0
FOXO3	F: CGGACAAACGGCTCACTCT R: GGACCCGCATGAATCGACTAT	19 21	61.9 61.7
FOXM1	F: ATACGTGGATTGAGGACCACT R: TCCAATGTCAAGTAGCGGTTG	21 21	60.3 60.3
METTL3	F: AGATGGGGTAGAAAGCCTCCT R: TGGTCAGCATAGGTTACAAG AGT	21 23	61.8 60.7
CHCHD2	F: ACACATTGGGTCACGCCATTA R: GCACCTCATTGAAACCCTCACA	21 22	62.0 62.7
IL33	F: GTGACGGTGTTGATGGTAAGAT R: AGCTCCACAGAGTGTTCCTTG	22 21	58 59
PRDX3	F: ACTGTGAAGTTGTCGCAGTCT R: CACACCGTAGTCTCGGGAAA	21 20	61.6 61.2
HTRA2	F: TTGCCATCCCTTCTGATCGTC R: TCAGCATCATCACCCCAATGTA	21 22	61.9 61.2
POLG	F: GAGAAGGCCCAGCAGATGTA R: ATCCGACAGCCGATACCA	20 18	60 60
RPLP0	F: TGCATCAGTACCCCATTCTATCA R: AAGGTGTAATCCGTCTCCACAGA	23 23	61 62

## Results

### Clinical investigation of ataxia telangiectasia patients

Demographic, clinical, imaging and immunological features of AT patients are summarized in Supplementary file: Table S1.

**Fig. 1:**
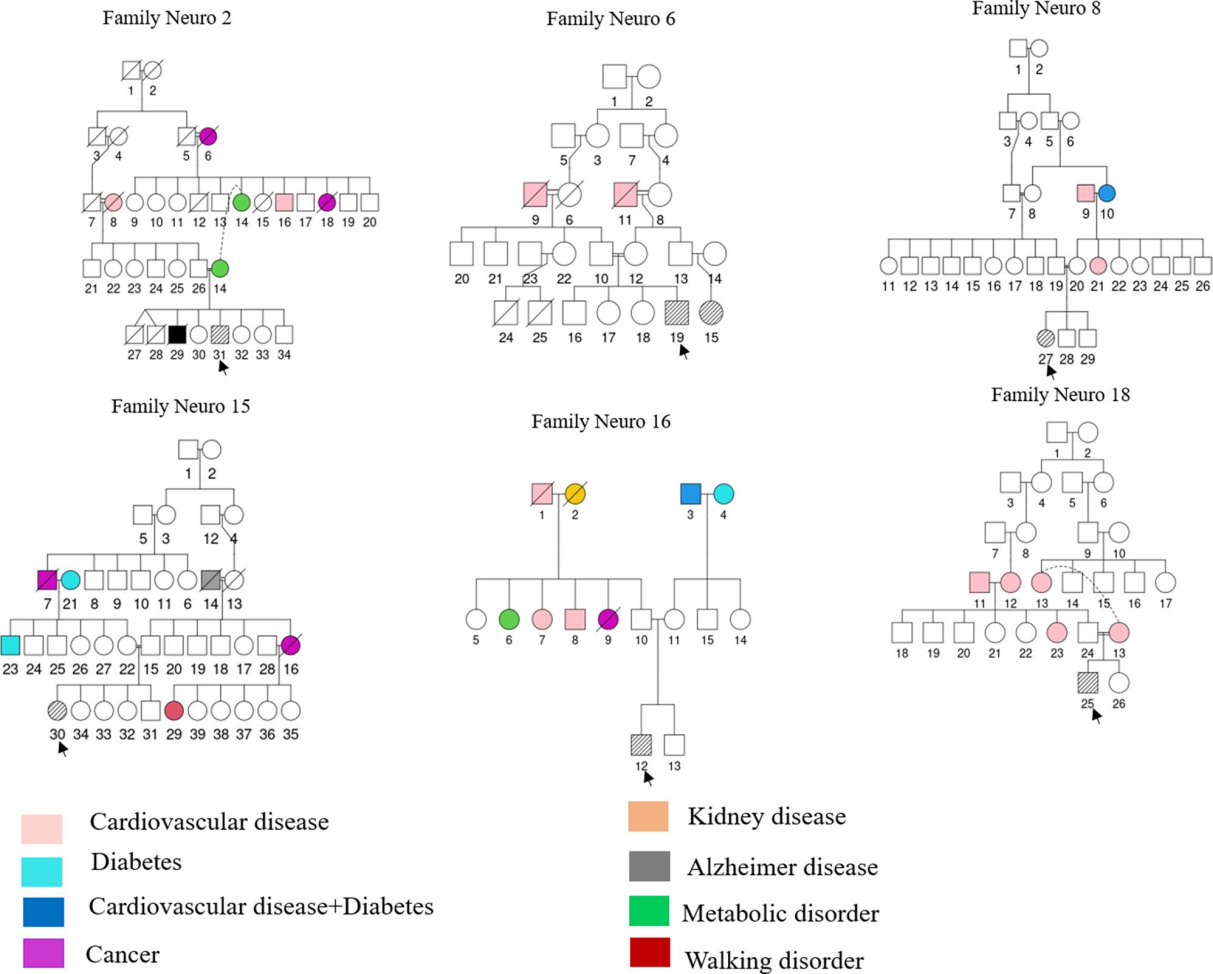
Pedigrees of Ataxia Telangiectasia patients families. Pedigrees correspond to six AT families. Arrows indicate probands. Filled symbols represent affected individuals according to the color code, open symbols represent unaffected individuals

Six AT patients were enrolled in the study; four males and two females. Five patients were born from consanguinous marriages, except for one patient. The pedigree of each family is presented in Fig. 1, indicating the affected individuals and showing relatives with other conditions and the degree of cansanguinity. The average age of patients was 13.3 years and the mean age of disease onset was 2.27 years.

Gait ataxia associated with postural instability and frequent falls were the first isolated revealing symptoms in all patients, except for one patient who presented speech impairment characterized by articulation difficulties, and tremor, prior to walking trouble. All patients presented static and kinetic cerebellar syndrome. The progressive worsening of gait was associated with impaired walking capacity preventing autonomous ambulation in all patients. Moreover, while only one patient showed pyramidal syndrome, extrapyramidal syndrome was recorded in all patients. Neuro8 patient presented a significant generalized frailty associated with muscle weakness in the lower limbs and pes cavus.

Intellectual disability and language delay were reported in two out of six patients. Cognitive memory deficits was also observed in Neuro8 patient. Limited intellectual efficiency and recent memory difficulties were documented in one patient. Two patients, notably Neuro2 and Neuro8 exhibited facial dysmorphic features.

Furthermore, ophtalmological abnormalities characterized mainly by telangiectasia and oculomotor apraxia, were noted in all patients. Strabismus was reported in Neuro8 patient only. Patient Neuro18 showed eye movement abnormalities characterized by impairment of saccadic pursuit.

Dermatological manifestations were documented in two patients.

Regarding immunological status, four patients were clinically immunodeficient, characterized by recurrent sino-pulmonary infections. Furthermore, lymphopenia was documented in one patient (Neuro15). The level of IgA was measured only in these four patients and the mean value was 0.13 g/l in those patients.

In addition, an elevated alpha-foetoprotein (AFP) level was recorded in the analyzed patients (five out of six) with a mean of 108.076 ng/ml.

Brain MRI has detected cerebellar atrophy in four out of six patients. Electrophysiological analysis revealed axonal polyneuropathy only in one patient. Additionally, abdominal ultrasound was performed for two patients (Neuro 15 and Neuro18) and has revealed a metabolic overload liver for both.

Cytogenetic analysis was performed for two patients and has uncovered a chromosomal translocation t [7, 14](p14,q11) in one of them.

### Mutational analysis of *ATM* and pathogenicity prediction

**Fig. 2:**
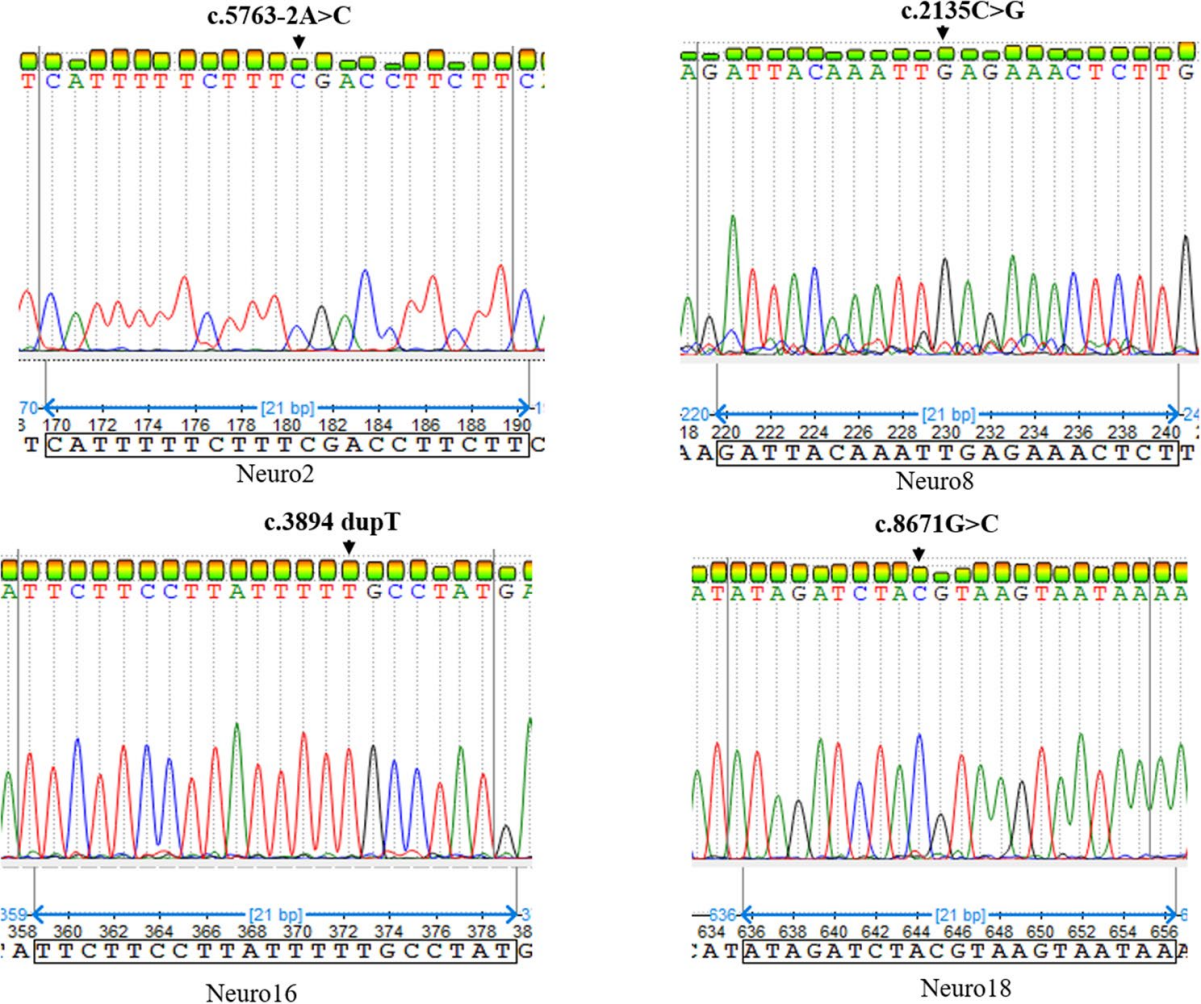
Electropherograms of ataxia telangiectasia patients. Electropherograms showing genetic variants in four AT patients. Arrow indicates the variant position in the nucleotide sequence

**Fig. 3:**
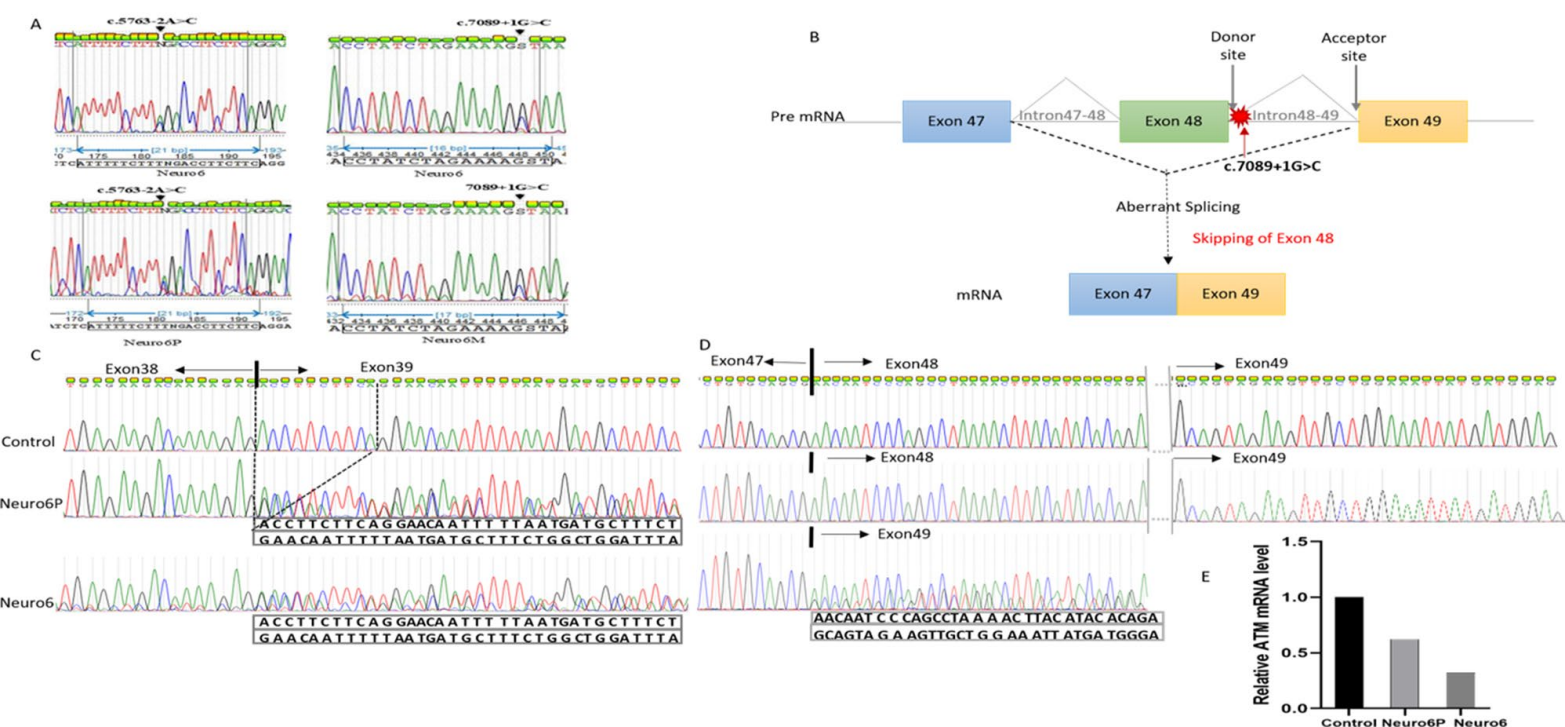
Sanger sequencing and RT-PCR for identification and functional characterization of splicing mutations in Neuro6 patient. (**A**) Electropherograms analysis showing mutations in Neuro6 patient heterozygous for c.5763–2 A > C and c.7089 + 1G > C and variants segregation among his parents. Arrows indicate the variant position. (**B**) Schematic representation of the predicted effect of c.7089 + 1G > C mutation (red star), showing exon48 skipping. (**C**-**D**) Sanger sequencing profile of cDNA illustrates the effect of the variants on the splicing for (**C**) c.5763–2 A > C (use of cryptic splice site 11nt downstream) and (**D**) c.7089 + 1G > C (exon48 skipping). Sequencing results are shown for control (top), Neuro6P (middle) and Neuro6 (bottom). (**E**) Relative ATM mRNA expression levels from PBMC of controls (*n* = 2), Neuro6P and Neuro6, data are expressed as fold change. Neuro6 corresponds to the proband, Neuro6P and Neuro6M correspond to father and mother of the patient

**Fig. 4:**
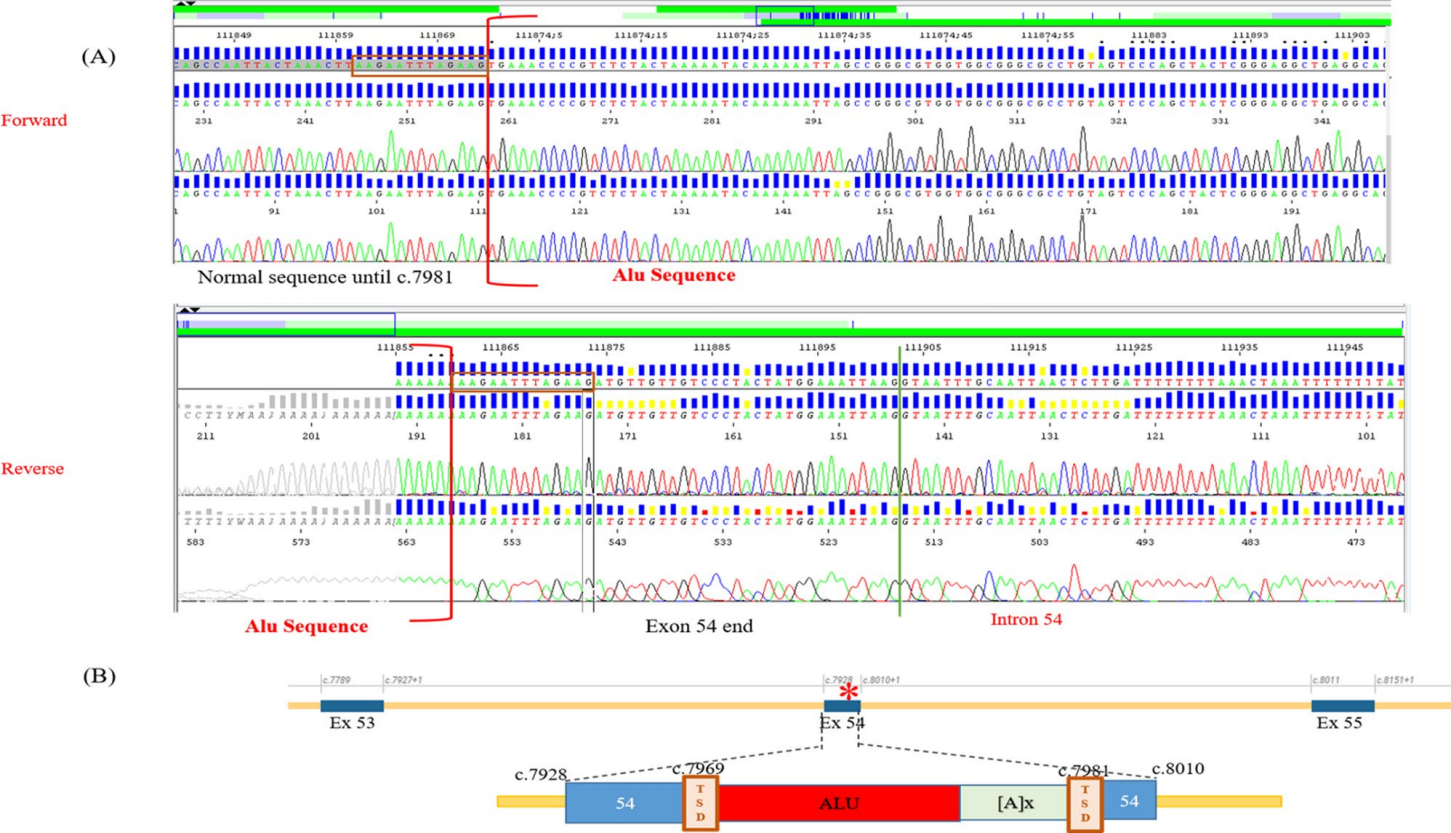
AluYb8 element insertion confirmed in Neuro15 patient. (**A**) Electropherograms from Neuro15 patient from forward and reverse sequences showing an Alu sequence insertion. The Alu sequence is delimited by brackets [.] in both orientations. TSD is highlighted in red box. (**B**) Schematic representation of ALuYb8 element insertion in ATM exon 54. TSD: Target Site Duplication. [A]x: poly(A) tail. The red star represents the mutation site

Genetic analysis using a gene panel identified six different mutations in the *ATM* gene, including two splice site mutations, two frameshifts and one missense mutation (Fig. 2). All included patients except Neuro6 were homozygous. In fact, Neuro6 patient was compound heterozygous. The segregation analysis showed that this patient has inherited c.5763-2A > C and c.7089 + 1G > C mutations from his father and mother, respectively, who were clinically healthy carriers (Fig. 3A). Neuro2 also harbored c.5763-2A > C mutation, but at homozygous state. Neuro8, Neuro16 and Neuro18 patients presented c.2135C > G (Ser712*), c.3894dup (Ala1299Cysfs*3) and c.8671G > C (Gly2891Arg) mutations, respectively. NGS analysis for Neuro15 patient expected an insertion of a retroelement in *ATM* gene at exon 54. This insertion was first checked using long-range PCR covering this region and subsequent PCR product gel electrophoresis, which was unsucceful (Supplementary file: Fig. S2). In order to confirm the presence of this element and to characerize it, a target PCR near the breakpoints was performed using different primer pairs flanking the insertion site followed by sanger sequencing. This confirmed the presence of a mobile element inserted into exon 54, as depicted in Fig. 4 (detailed information are presented in the supplementary file: Supplemental data). The identified sequence corresponded to a 5’-truncated *Alu*Yb8 element based on Dfam alignement with one mismatch (Supplementary file: Fig. S3). Sanger sequencing revealed an insertion of AluYb8 element at chr11(GRCh38):g.108333939_108333940 insAluYB8; NM 000051:c.7981_7982insAluYb8. A target site duplication of 13 bp (5’-AAGAATTTAGAAG-3’) was identified. The core Alu sequence was of 188 bp and the poly(A)-tail was estimated to be 25pb. Indeed, the length of the poly(A)-tail could not be determined with certitude due to polymerase slippage during amplification. The sequences of different primers used for this analysis are represented in Supplementary file: Table S2. Alu element insertion was predicted to disrupt ATM coding sequence and to induce a premature stop codon six residues downstream the insertion, possibly generating a truncated ATM protein (p.Asp2661Valfs*7) (Supplementary file: Fig. S4**)**.

The c.3894dup frameshift mutation was located in exon 26 and has been previously documented. It creates a stop codon after three amino acid from the mutation site (Ala1299Cysfs*3). It was predicted by NMDEscPredictor tool to cause protein degradation through a non-sense-mediated mRNA decay machinery.

Among identified variants, the c.7089 + 1G > C mutation has not been reported before according to the gnomAD and ExAC databases. Furthermore, we described, for the first time to our knowledge, the c.8671G > C mutation in patient with AT disease, previously reported in T-cell prolymphocytic leukemia (T-PLL) [43]. Indeed, the c.7089 + 1G > C and c.8671G > C mutations were located in intron 48–49 and exon 59, respectively. All identified variants and their corresponding ACMG/AMP classifications are summarized in Supplementary file: Table S3. The c.7089 + 1G > C and c.8671G > C mutations were predicted as deleterious by Mutation Taster tool and pathogenic by FATHMM. The c.8671G > C and c.7089 + 1G > C mutations were classified by Clinvar as variants with uncertain significance and likely pathogenic, respectively. The c.7089 + 1G > C mutation was predicted as pathogenic by CADD, and the c.8671G > C was predicted as pathogenic by CADD and REVEL and as disease-associated with high confidence (RI = 9) by PhD-SNP. Conservation analysis using the ConSurf tool showed that this variant affects a highly conserved residue; displaying a conservation score of 9, which highlights the relevance of mutation at this site (Supplementary file: Fig. S5)*.*

The splice site variant c.7089 + 1G > C was predicted by HSF and SpliceAI tools to likely disrupt the canonical splicing by causing the loss of the donor splice site, with a delta score of donor loss = 1. According to Varseak tool, the c.7089 + 1G > C mutation was predicted to induce the loss of function for an authentic 5’ splice site and to exon 48 skipping (Class 5 splicing effect) (Fig. 3B). Furthermore, Neuro6 patient was also carrier of c.5763–2 A > C mutation, which we have previously reported it in other AT patients, and was predicted to affect splicing process by inducing a loss of function of the splice site and potentially the use of cryptic acceptor site 11 nucleotides downstream of the original site [44]. The predicted effects on the splicing were confirmed for both splice site mutations through RT-PCR followed by sanger sequencing, and aberrant ATM transcripts were detected in Neuro6P and Neuro6 carriers of one or the two mutations, as represented in Fig. 3C, D. Additionaly, in order to assess the effect of mutations on total ATM mRNA expression level, a qPCR was conducted for Neuro6 patient and his father Neuro6P. Relative expression analysis of *ATM* was carried out using the 2⁻ΔΔCt method, with healthy controls set as baseline (fold change = 1). Results showed that ATM mRNA level was decreased in both individuals, and was markedly reduced in Neuro6 relative to healthy controls, with a fold change of 0.319 in Neuro6 patient and 0.6236 in Neuro6P, which corresponds to a reduction by approximately 68% and 38%, respectively, relative to healthy controls (Fig. 3E).

Subsequently, the Expasy tool was explored to predict the impact of this splicing mutation at protein level. According to this tool, exon 48 skipping leads to an in-frame deletion of amino acids from Asparagine at position 2326 to Lysine at position 2363 (p. Asn2326_Lys2363 del). In the other side, to infer the potential effect of c.8671G > C (Gly2891Arg) mutation at the protein level, I-mutant and MuPro tools were employed and have predicted that this variant resulted in a slight decrease of protein stability (∆∆G = -1.83, ∆∆G = -0.87023447 for I-Mutant and MuPro, respectively). MutPred tool predicted a pathogenic effect with a g score of 0.878. To better predict the effect of c.8671G > C (Gly2891Arg) mutation on the native protein structure, it was further subjected to HOPE and Missense3D analyses, using the wild-type PDB ID 7SIC as a template structure. Missense3D tool predicted structural damage induced by Gly2891Arg mutation. This could be explained by a contraction of the cavity volume by 211.896 Å3 as a result of glycine to arginine substitution. In addition, the project HOPE tool indicated that the mutant and the wild-type residues present different physicochemical properties. Indeed, arginine is a big, less hydrophobic and positively charged amino acid compared to glycine which is a small, hydrophobic and neutral amino acid. Glycine is a highly flexible protein, allowing it to adopt torsion angles. The substitution of a glycine with an argine, a larger and more rigid residue, could force the local backbone into an unfavorable conformation, leading to structural disturbances. Moreover, the larger size of arginine may introduce steric clashes, further destabilizing the protein structure.

### Bioinformatic analysis

To further evaluate the putative effect of the novel identified missense mutation on ATM protein function through its interaction with ATP and its structural flexibility, bioinformatic analysis was carried out.

#### Molecular docking

**Fig. 5:**
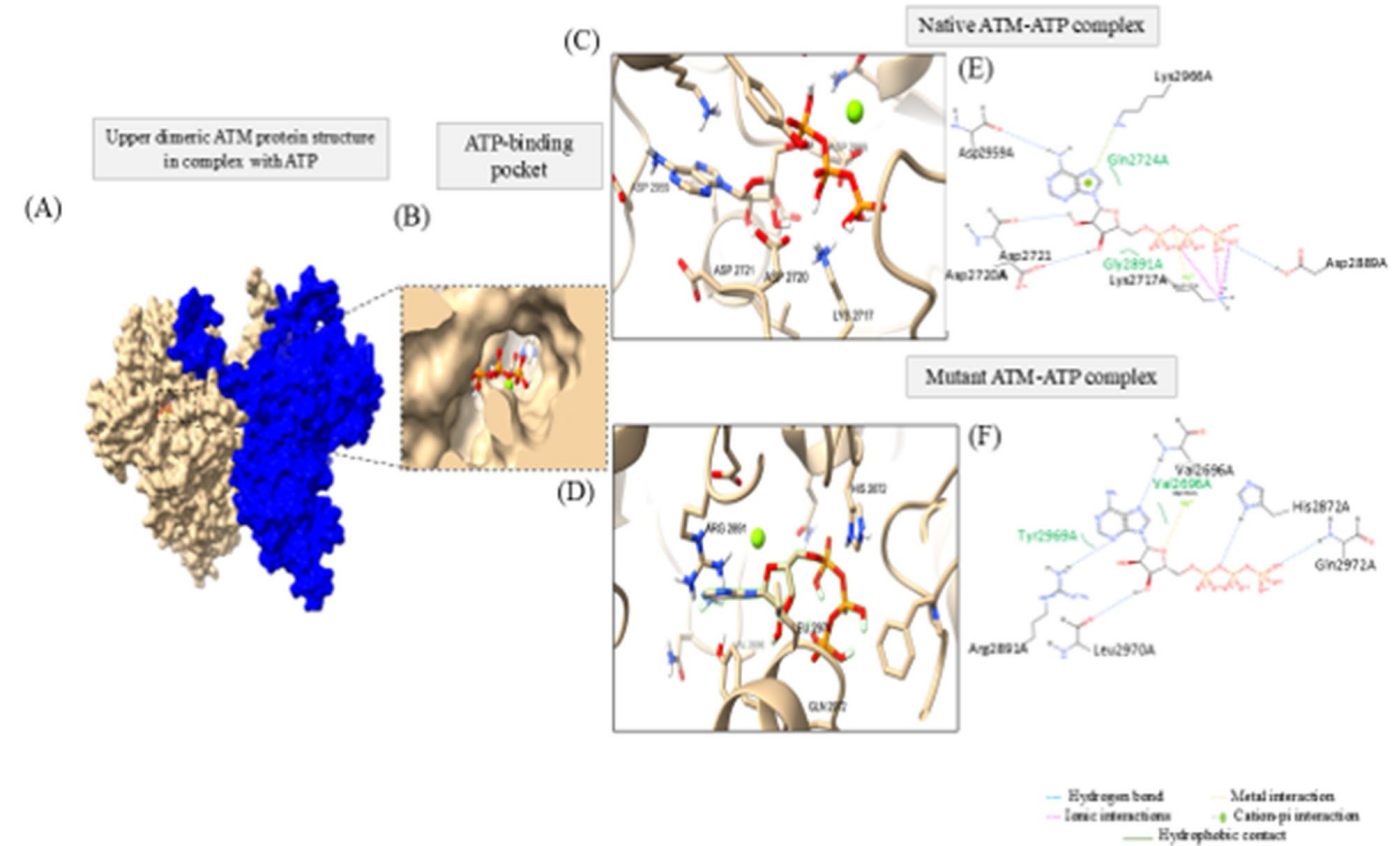
Molecular docking results of ATM and ATP interactions. (**A**) Upper ATM dimeric structure in interaction with ATP. (**B**) ATP binding pocket within ATM protein. (**C**-**D**) Enlarged view of 3D; and (**E**, **F**) 2D intermolecular interactions between ATM native or mutant structure and ATP, using ChimeraX and PoseEdit tools, respectively. Colors correspond to the interaction types as indicated by the color key shown in the figure. Mg atom is shown as green sphere

In order to assess the functionnal consequence of Gly2891Arg mutation on ATP binding affinity, a molecular docking analysis was conducted using upper dimeric ATM native or mutant structure as receptor and ATP as ligand in the presence of Mg atom (Fig. 5). Figure 5A, B depict ATP binding within ATM binding pocket.

Indeed, the Gly2891Arg variant showed relatively lower binding affinity toward ATP compared to wild-type protein (-7.073 vs. -7.645). The analysis of the binding mode showed that the mutant structure displayed different interaction pattern compared to the wild-type, in terms of numbers, types of interactions and residues involved, as illustrated in Fig. 5C, D. While mutant complex showed only H bonds interactions with His2872, Gln2972, leu2970, Val2696 and Arg2891, the wild-type presented H bonds with Lys217, Asp2720, Asp2721 Asp2959, ionic interactions with Lys2717 and cation-pi interaction with Lys2966.

#### Molecular dynamic simulation

To analyze the effect of mutation on protein dynamics, the flexibility of both native and mutant ATM protein structures was assessed by CABS-flex 2.0 software. The RMSF (Root Mean Square Fluctuation) plots presented the residual fluctuation of the structure models associated with a decreased values from wild-type (0.0530 to 10.0760 Å) to mutant protein (0.0470 to 5.51570 Å), consistent with a less flexible mutant protein than the wild-type (Supplementary file: Fig. S6). Specifically, the average RMSF value of activation loop residues (A2888-A2911) was 1.292Å for the wild-type ATM protein compared to 0.97625 for the mutant protein structure. The decrease of RMSF values upon Gly2891Arg mutation reflected a change in protein dynamic behavior altering structural flexibility associated with a more rigid protein structure.

### Immunological profilinig in patients with ataxia and in their relatives

Following the identification of genetic variants, we thought to assess the immune profiling of AT patients, their parents and patients with other forms of ataxia. Furthermore, we attempted to explore the relationship between ATM mutational type and immunological phenotype in AT patients.

### LB cells

**Fig. 6:**
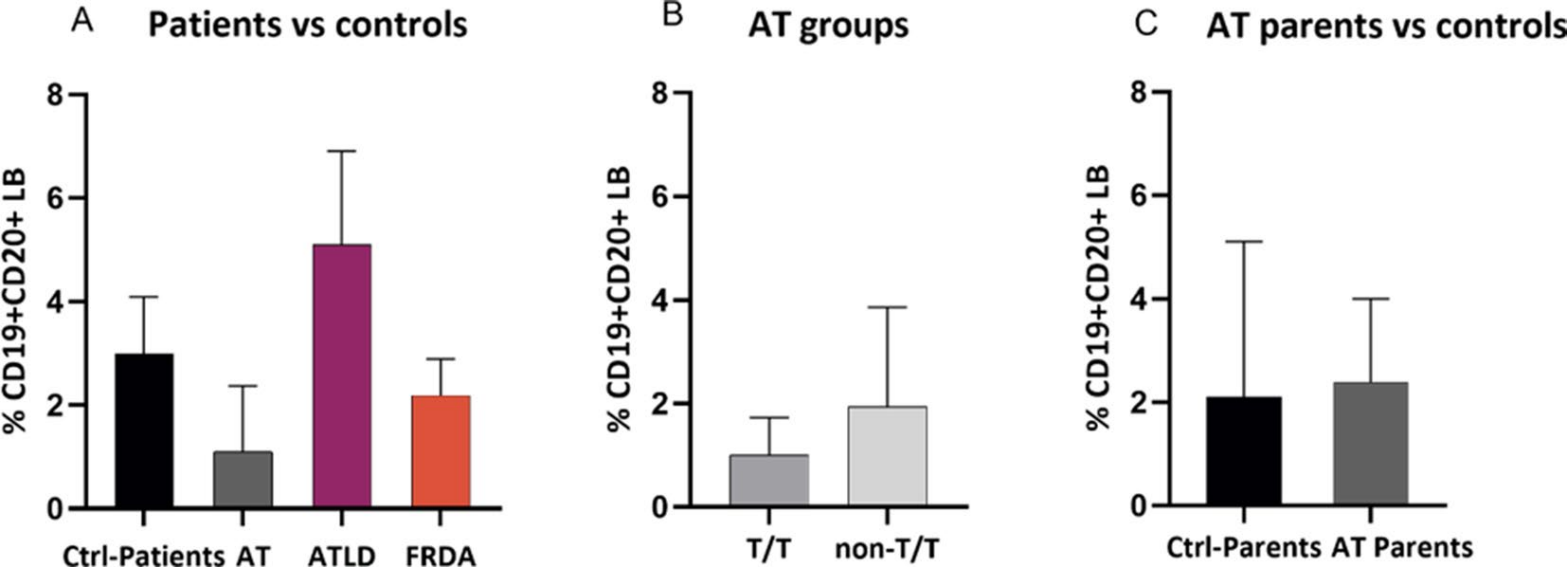
Analysis of LB cells distribution across different groups. (**A**) Percentages of LB cells in AT (*N* = 9), ATLD (*N* = 2) and FRDA (*N* = 2) patients compared to age-matched healthy donors (*N* = 3); (**B**) among different AT groups depending on their genotype (T/T: *N* = 5 vs. non-T/T: *N* = 4); and (**C**) in AT parents (*N* = 6) compared to age-matched healthy donors (*N* = 6). Data are presented as median ± IQR. N = number of subjects

The percentage of LB cells was lower in AT (1.1 ± 1.425) compared to healthy young donors (3 ± 2.86), while it was relatively similar in ATLD1 (5.1 ± 3.62) and FRDA patient groups (2.19 ± 1.42) compared to healthy controls (Fig. 6A). We further assessed the distribution of LB cells in AT patients depending on their mutational type, a trend toward a slightly decreased percentage was observed in T/T (1 ± 1.04) group compared to non-T/T group (1.94 ± 2.81) (Fig. 6B). All patients included in T/T groups present a reduced value, whereas one out of four patients from non-T/T group presented an elevated value compared to the mean value of healthy donors. Additionally, the immunophenotypic analysis of AT patients’ parents showed no difference in the percentages of LB cells, in AT parents (2.39 ± 3.6) and in age-matched healthy donors (2.1 ± 3.347) (Fig. 6C).

### LTCD4 + and LTCD8 + cells

**Fig. 7:**
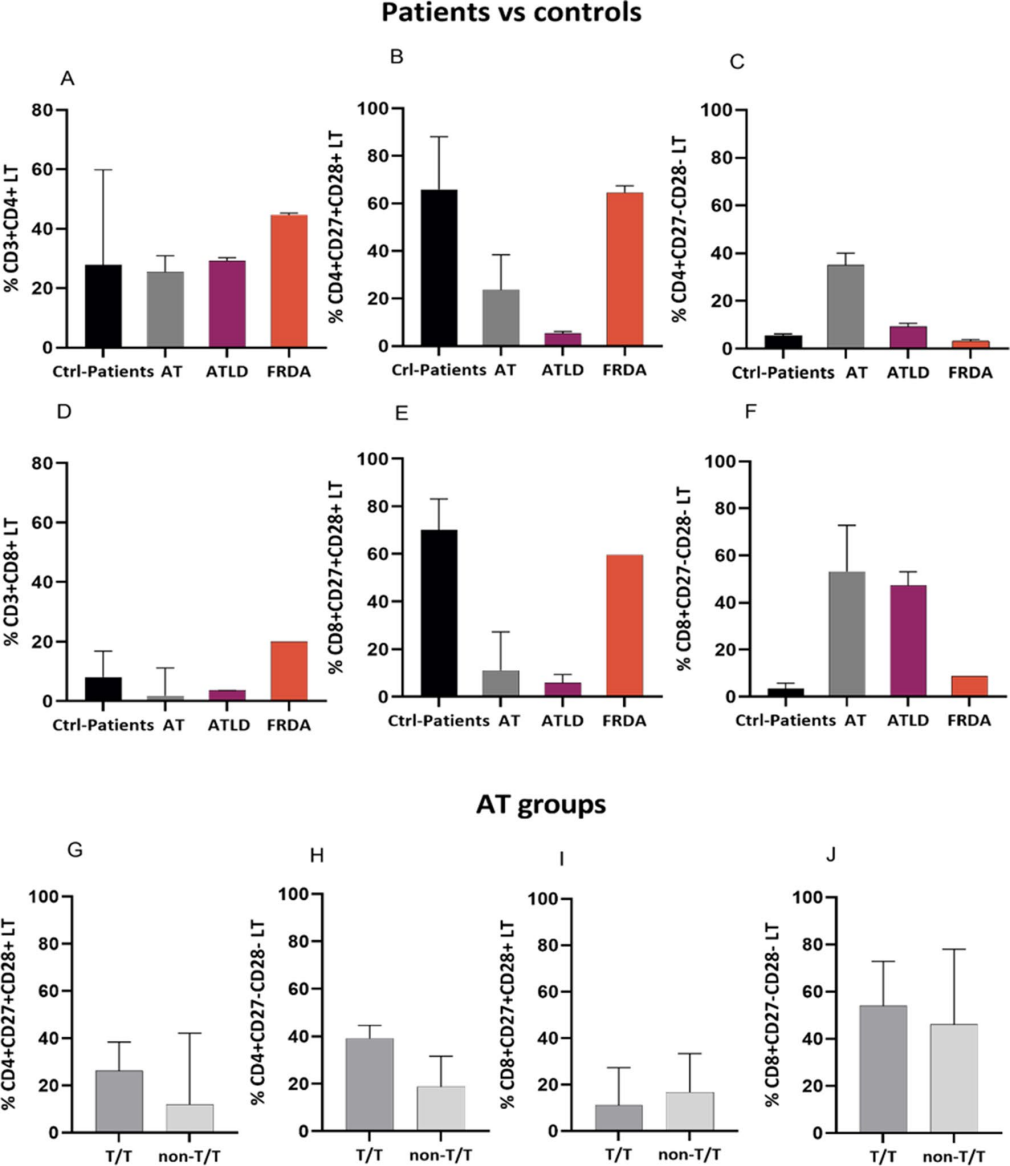
Analysis of LTCD4 + and LTCD8 + subsets distribution across different groups. (**A**-**C**) Percentages of total and subsets of LTCD4+; (**D**-**F**) total and subsets of LTCD8 + in AT (*N* = 6), ATLD1 (*N* = 2) and FRDA (*N* = 2 for LTCD4 + and *N* = 1 for LTCD8+) patients compared to healthy donors (*N* = 6); and (**G**-**J**) among different AT groups depending on their genotype (T/T: *N* = 5 vs. non-T/T: *N* = 4). Data are presented as median ± IQR. N = number of subjects

No difference in the percentage of total LTCD4 + was noted in ARCA patients compared to healthy donors, while a notable decrease of total LTCD8 + was observed in AT patients compared to healthy donors (1.81 ± 10 vs. 8.1 ± 12.3) (Fig. 7A, D). Although this overall reduction of LTCD8 + cells in the majority of patients, two patients out of five harboring a T/T genotype; notably with c.2135 C > G (Ser712*) or c.3894dup (Ala1299Cysfs*3) mutations showed an increase of this cell population compared to healthy donors. A decreased percentage of LTCD4 + in T/T group compared to non-T/T group was observed (17.3 ± 23 vs. 27.2 ± 6.8), with four out of five patients in T/T group present values lower than the mean value in healthy donors, while non-T/T group present comparable values. Next, the senescence of T cells was assessed. The gating strategy was set to identify senescent cells based on their loss of CD27 and CD28 expression. In fact, changes of T cells subsets were more prominent in LTCD8 + compared to LTCD4 + in AT patients. Indeed, AT patients showed a tendency toward a decrease of LTCD4 + CD27+CD28+ (23.6 ± 34) compared to healthy donors (65.9 ± 23.172.37), alongside an increase of senescent LTCD4 + CD27-CD28- (35 ± 21 vs. 5.6 ± 5.04) (Fig. 7B, C). Furthermore, AT patients exhibited a reduced percentage of LTCD8 + CD27+CD28 + subtype (11.1 ± 21.83 vs. 70 ± 18.4) accompanied with an increased percentage of LTCD8 + CD27-CD28- senescent subset (53.2 ± 32.2 vs. 3.4 ± 2.56) (Fig. 7E, F). While no obvious difference was found for both subsets in FRDA patients compared to healthy donors, the percentages of activated LTCD4 + CD27+CD28 + and LTCD8 CD27 + CD28+ cells appeared to be lower in ATLD1 patient group (15,04 ± 18,19 vs. 72.37 ± 12.08) for LTCD4 + and (5.4 ± 1.5 vs. 70 ± 18.4) for LTCD8, respectively. A notable increase of senescent LTCD8 + cells was also detected (47.25 ± 11.5 vs. 3.4 ± 2.56) in ATLD1 patients. We further assessed the distribution of LTCD4 + and LTCD8 + subsets within the AT patients’ group. Regarding LTCD4 cells, our results showed an elevated proportion of LTCD4 + CD27-CD28- in T/T group compared to non-T/T AT group (39.1 ± 11.9 vs. 18.8 ± 18), and unexpectedly the T/T group displayed a higher percentage of LTCD4 CD27 + CD28+ (26.2 ± 24 vs. 11.84 ± 38) (Fig. 7G, H). Although most patients tended to show a shift toward a senescent phenotype characterized by an elevation of LTCD4 + CD27-CD28- subsets over LTCD4 + CD27+CD28- cells, one patient in each of the groups exhibited the opposite pattern, yet both subsets remain consistent with the overall trend relative to healthy donors (Fig. 7G, H). For LTCD8+, independently of ATM mutational type, both AT patient groups exhibited a reduced LTCD8 + CD27+CD28 + and an elevated LTCD8 + CD27-CD28-, which was modestly more prominent in T/T group compared to non-T/T group (11.1 ± 21 vs. 16.7 ± 29.6 for activated LTCD8 + CD27+CD28 + cells, and 53.9 ± 27.2 vs. 46.1 ± 38 for senescent LTCD8 + CD27-CD28-cells) (Fig. 7I, J).

**Fig. 8:**
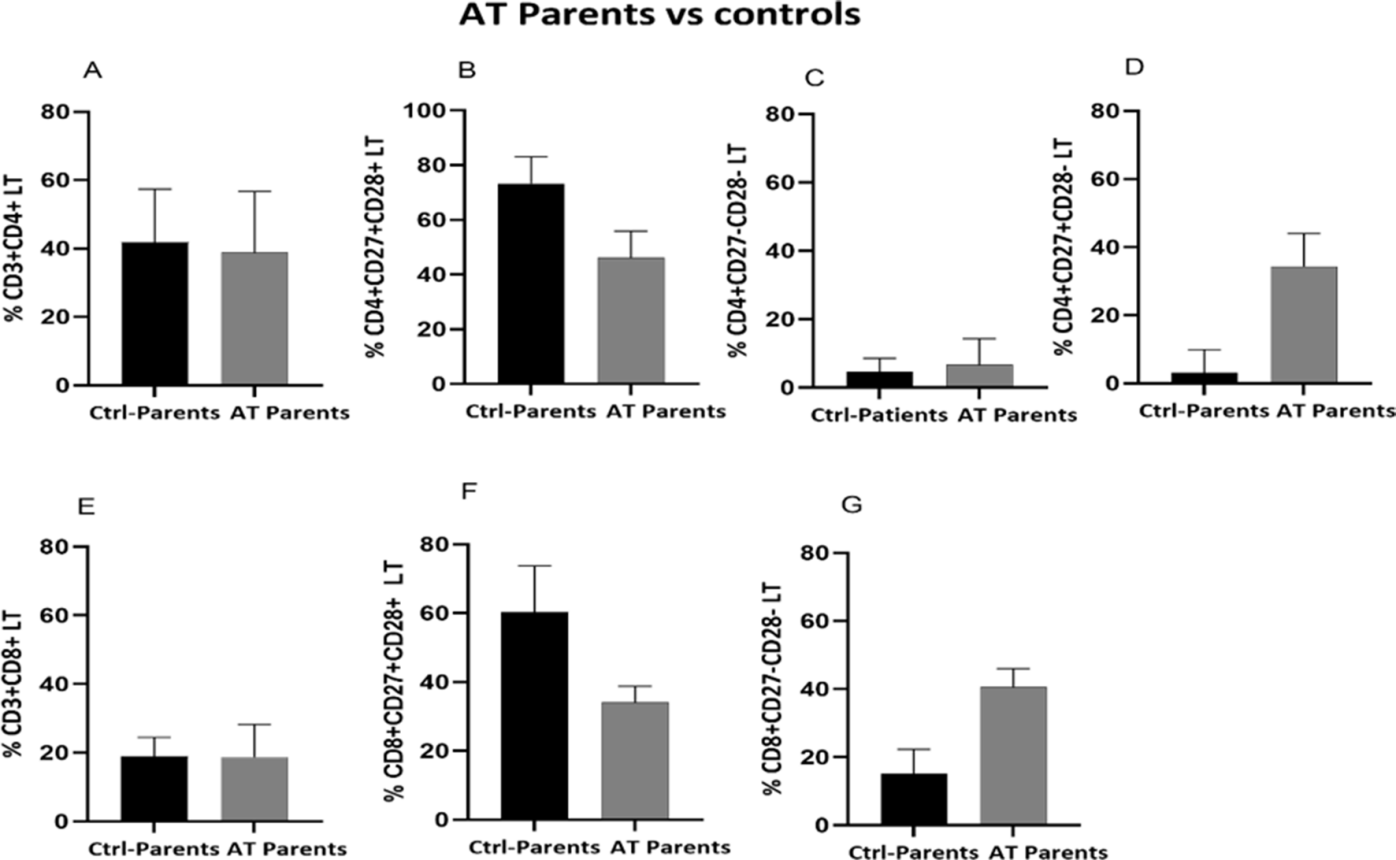
Analysis of LTCD4 + and LTCD8 + subsets distribution in AT parents compared to healthy donors. (**A**-**D**) Percentages of total LTCD4 + and their subsets of; (**E**-**G**) total LTCD8 + and their subsets of in AT patients (*N* = 6) compared to healthy donors (*N* = 6). Data are presented as median ± IQR. N = number of subjects

Additionally, no difference in LTCD4 + and LTCD8 + percentage was noticed between AT parents (38.8 ± 35 and 18.70 ± 27.4, for LTCD4 + and LTCD8+, respectively) and healthy donors (41.75 ± 20.13 and 18.90 ± 16.172 for LTCD4 + and LTCD8 + cell subsets, respectively), as demonstrated in Fig. 8A, E. Nevertheless, AT parents exhibited a similar distribution of different LTCD8 + cell subsets mimicking their children characterized by a decreased LTCD8 + CD27+ CD28 + subset (34 ± 15.7 vs. 60.3 ± 28.23) and an increased LTCD8 + CD27-CD28- cells (40.6 ± 15.2 vs. 15.1 ± 13.29) compared to age-matched healthy donors (Fig. 8F, G). While the percentage of LTCD4 + CD27+CD28 + seemed to decrease in AT parents (46 ± 13.25) compared to age-matched healthy donors (73.3 ± 18.03), the percentage of LTCD4 + CD27-CD28- was not different from their healthy counterparts (Fig. 8B, C). To better understand this distribution of LTCD4 subsets, we have assessed the loss of CD28 marker expression, our analysis revealed an apparent expansion of LTCD4 + CD27+CD28- subpopulation in AT parents (34.35 ± 18.85 vs. 3.13 ± 9.33 ± 9.3) (Fig. 8D).

### NK and NKT cells

**Fig. 9:**
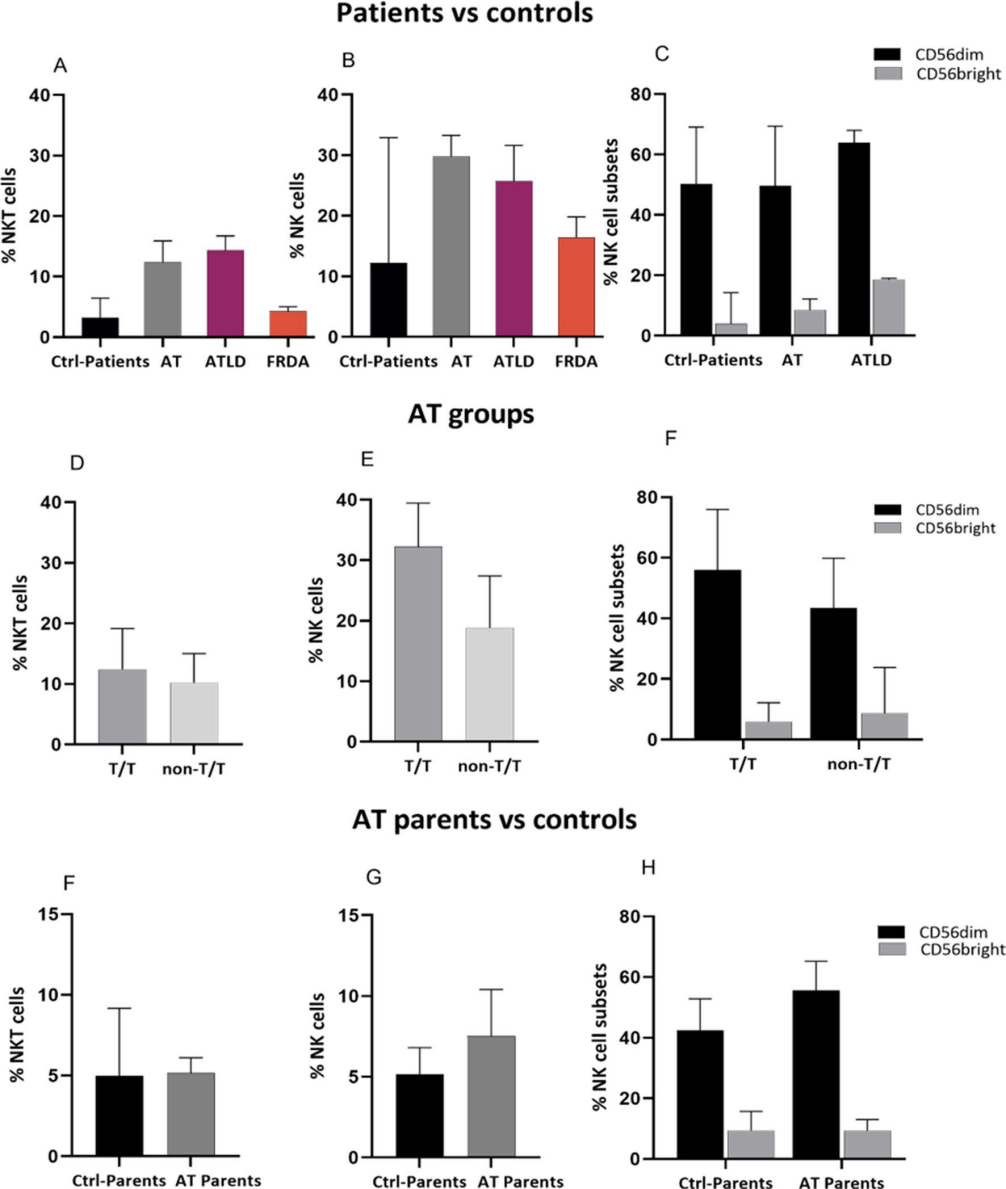
Analysis of NKT and NK cells distribution across different groups. (**A**-**C**) Percentages of NKT and NK cells in AT (*N* = 9), ATLD1 (*N* = 2) and FRDA (*N* = 2) patients compared to age-matched healthy donors (*N* = 3); (**D**-**F**) in different AT patient groups depending on their genotype (T/T: *N* = 5 vs. non-T/T: *N* = 4); and (F-H) in AT parents (*N* = 6) compared to age-matched healthy donors (*N* = 4). Data are presented as median ± IQR. N = number of subjects

The percentages of total NKT and NK cells seemed to be higher in AT (12.40 ± 7.845 and 29.8 ± 14.5 for NKT and NK respectively) and ATLD1 (14.4 ± 4.6 and 25.75 ± 11.7) patients compared to healthy donors (3.2 ± 3.74 and 12.2 ± 27.36), whereas the FRDA patients group showed values comparable to healthy donors’ group (4.295 ± 1.43 and 16.45 ± 6.7) (Fig. 9A, B). Based on AT patients’ genotype, both subsets appeared higher in T/T group compared to non-T/T group relative to healthy controls, with NK cells were markedly higher (32.2 ± 11.1 vs. 18.8 ± 14.65 for T/T and non-T/T genotype group, respectively) (Fig. 9D, E). Compared to healthy donors, all patients with T/T genotype presented a notably higher values of NK and NKT cells percentages, while three out of four patients in non-T/T group presented relatively more elevated percentage of these cells. Furthermore, the proportions of two major NK cell subsets defined by cell maturation status; CD56dim mature subset and CD56 bright immature subset, that represent the predominant and the minor fractions of NK cells, respectively, were evaluated in AT and ATLD1 patients relative to healthy-donors. Results shown a similar distribution of subsets, with a slight increase of CD56dim and CD56bright in ARCA patient groups compared to age-matched healthy donors (Fig. 5C). To further assess the distribution of NK cell subsets among AT and ATLD1 patient groups, CD56 dim NK cells were divided based on their CD57 marker expression and the CD57negNK/CD57pos NK ratio was subsequently determined. A trend toward a decreased ratio was observed in patients compared to healthy donors (0.37 ± 0.6104 and 0.3994 ± 0.1251vs 0,9006 ± 1.2243 for AT, ATLD1 and healthy controls groups, respectively).

The proportions of NKT and NK cells were comparable between AT parents and age-matched healthy donors. Additionally, similar distribution pattern of CD56dim and CD56bright NK cells was also observed between both groups (Fig. 9F-H).

### Monocytes

**Fig. 10:**
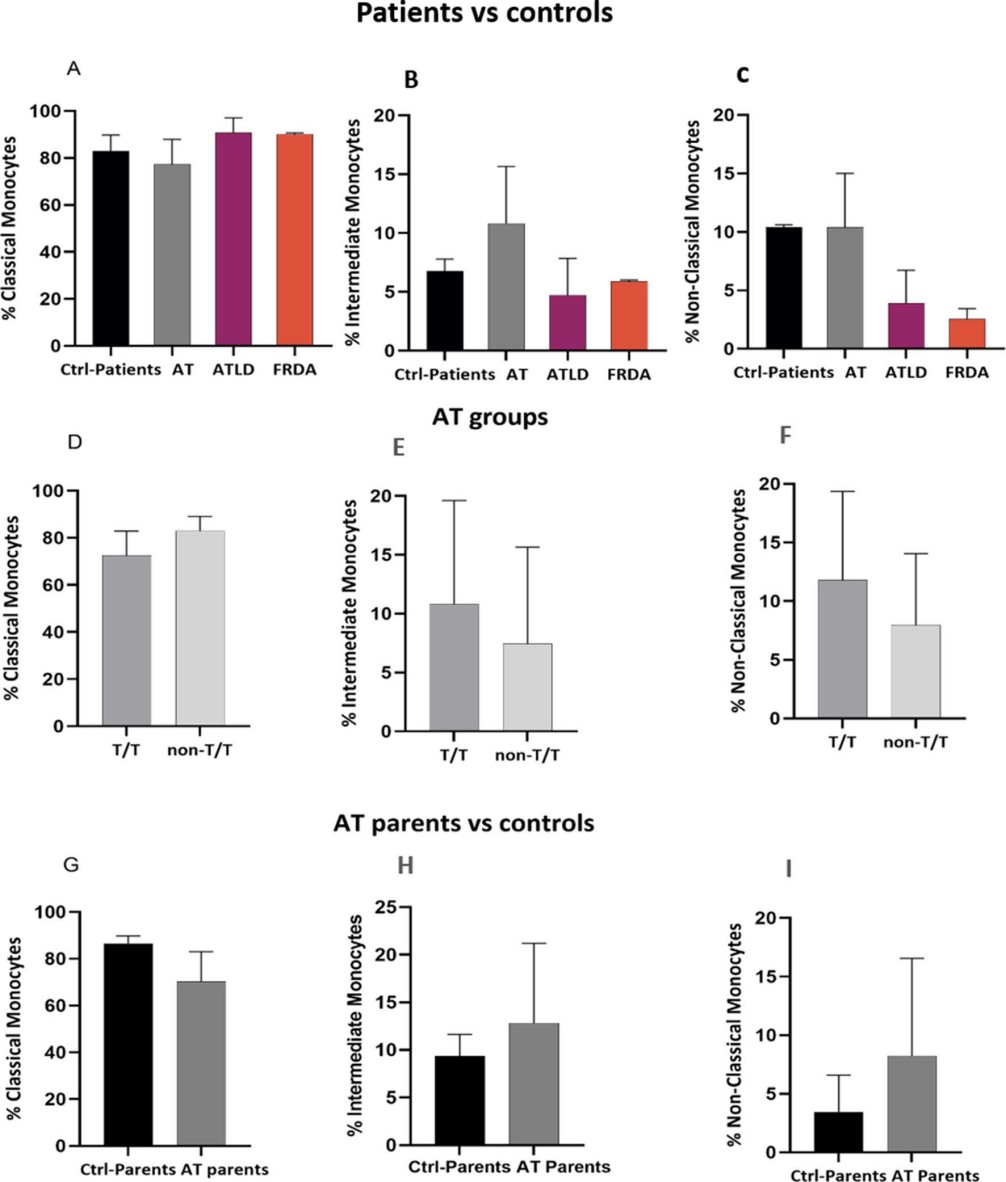
Analysis of monocyte subsets distribution across different groups. (**A**-**C**) Distribution of monocyte subsets in AT (*N* = 9), ATLD1 (*N* = 2) and FRDA(*N* = 2) patients compared to age-matched healthy donors (*N* = 3); (**D**-**F**) in AT patient groups (T/T: *N* = 5; non-T/T: *N* = 4); and (**G**-**I**) in AT parents (*N* = 4) compared to age-matched healthy donors (*N* = 6). Data are presented as median ± IQR. N = number of subjects

The analysis of the percentage of monocyte subsets revealed an observed relative difference of monocyte distribution with a slight decrease in the proportion of classical monocytes and an increase of intermediate monocytes, while non classical monocytes remain unchanged in AT patients compared to healthy controls, with a percentage of 77.3 ± 25.6 vs. 83.00 ± 8.6, 10.8 ± 8.405 vs. 6.78 ± 1.41 and 10.4 ± 11.5 vs. 10.4 ± 7.21, respectively. Conversely, the proportions of different subsets in ATLD1 (90.90 ± 12.4; 3.890 ± 5.66; 4.715 ± 6.25, for classical, non-classical and intermediate monocytes, respectively) and FRDA (90.15 ± 1.1; 2.540 ± 1.8; 5.870 ± 0.28) patient groups were within the normal range (Fig. 10A, B, C). Difference between AT group and healthy controls was notable in T/T genotype (72.50 ± 22.25; 11.8 ± 12.415; 10.8 ± 11.44, for classical, non-classical and intermediate monocytes, respectively) whereas the overall proportions of monocyte subsets in non-T/T group seemed to be more similar to healthy controls (83.05 ± 23.95; 5.740 ± 12.55; 11.12 ± 8.932) (Fig. 10D, E, F). Regarding classical monocytes; four out of five patients from T/T group displayed lower percentage than healthy donors, while two out of four patients in non-T/T group showed this decrease. Although for intermediate monocytes, all patients independently of their *ATM* mutational type, harbored an increased proportion of intermediate monocytes compared to healthy donors but at different extent, three patients out of five and only one out of four from T/T and non-T/T AT patient group, respectively, presented an increase in non-classical monocyte subsets.

In the other side, AT parents displayed a relatively similar pattern to affected AT patients. They presented a decrease in classical monocytes, with an increase of both non-classical and intermediate monocyte subsets compared to healthy donors (70.25 ± 27.2 vs. 86.6 ± 12.65; 8.245 ± 9.442 vs. 3.45 ± 4.375 and 12.85 ± 14.48 vs. 9.36 ± 7.517, for classical, non-classical and intermediate monocytes in AT parents vs. healthy donors, respectively) (Fig. 10G, H, I).

### Gene expression analysis

Besides cellular impairments of peripheral immune cells, we tried to explore molecular alterations reflected by transcriptional changes in these cells in relation to clinical severity in AT patients.

Gene expression analysis was conducted for a set of candidate genes involved in cellular stress-response related pathways and mitochondrial function.

**Fig. 11:**
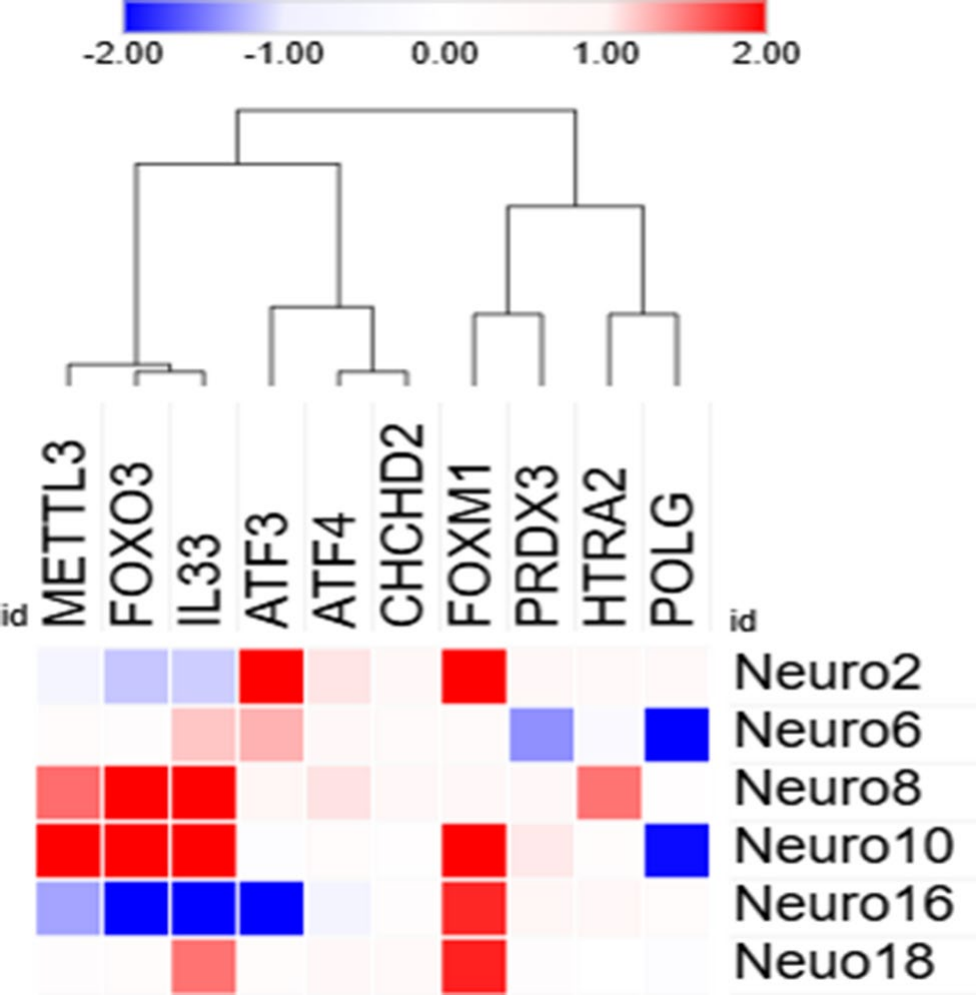
Hierarchically clustered heatmap depicting gene expression changes in AT patients using Spearman rank correlation Heatmap shows relative gene expression as log2FC. Rows represent patients and columns represent genes. Red indicates upregulated gene and blue represents a downregulated gene, with color intensity being proportional to the values, while neutral/white color indicates a non- differentially expressed genes. The columns were presented based on hierarchical clustering

We observed differences in gene expression profiles between patients that could explain clinical phenotypic variability among AT patients (Fig. 11). An overexpression of *FOXM1* has been noted in four out of six patients (Neuro2, Neuro8, Neuro10 and Neuro18). *ATF3* was also upregulated in two patients (Neuro2 and Neuro6). While hierarchical clustering was unable to classify patients into groups according to their expression patterns, a shared expression profile of three genes, notably *IL33*, *FOXO3* and *METTL3*, in Neuro8 and Neuro10 patients was recorded.

## Discussion

In this study, we aimed to conduct a genetic and immunological characterization of AT Tunisian patients, and to explore candidate gene expression pattern which may provide further insights into disease pathogenesis. To the best of our knowledge this is the first immunophenotyping study conducted in AT Tunisian patients, and in AT patients’parents. In addition, we reported the first peripheral immune profiling of clinically suspected ATLD1 and FRDA patients. Our findings have identified three novel mutations which widens the spectrum of *ATM* causative pathogenic variants in AT disease worldwide. The pathogencity of theses variants were assessed by cDNA analysis and computationnal study. Our study confirm the pathogenic effect of splicing mutations on the transcript sequence and on *ATM* expression. Bioinformatic analysis suggested that the novel identified missense mutation could probably affect ATM-ATP interaction pattern and protein flexibility. Our study described also a novel insertion of an Alu sequence in *ATM* gene, at exon 54, that probably induces a premature stop codon likely resulting in a truncated protein. Furthermore, immune cell phenotyping of AT patients’parents identified immunological defects that could be partly associated with health risks linked to *ATM* heterozygosity. Finally, our analysis identified three candidate genes that seem to be associated with AT disease progression and clinical severity.

FRDA and AT are the most frequent forms of ARCA, wherease ATLD1 is a very rare form with a few cases reported worldwide. AT represent the most common genetic ataxia with early onset [45]. They remain undiagnosed in Tunisia, a country with a high rate of consanguinity and endogamy.

### Clinical and genetic characterization

All patients in our cohort showed static and kinetic cerebellar syndrome as well as an extrapyramidal syndrome. Another cardinal feature of AT disease is immunodeficiency, which was detected in the majority of patients.AT is a prototype of DNA repair disorders, caused by biallelic mutations in the *ATM* gene encoding ATM protein, which is a master regulator of DNA damage response (DDR) [46]. Mutations in *ATM* are spread along the gene with no hotspots. Extensive research efforts have been devoted to identifying genetic variants in AT disease [47–49]. To date, more than 1400 unique mutations have been identified in *ATM* gene [50]. The majority of *ATM* mutations are null mutations resulting in the absence of the protein and/or its kinase activity. Missense or leaky splice site mutations are also reported and may result in retained ATM kinase activity [51]. Our genetic screening has identified seven different mutations, three have not been previously reported in AT disease. In fact, c.5763–2 A > C and c.2135 C > G (ser712*) mutations have been recently reported in AT Tunisian patients in our previous study [52]. According to the litterature, c.5763–2 A > C and c.2135 C > G (Ser712*) have been described previously in other AT patients [52, 53]. The c.2135 C > G (Ser712*) mutation has been in associated with a severe clinical AT phenotype [41, 54] and was documented in breast cancer [55]. This was in accordance with our cohort, in which Neuro8 carrying this mutation in homozygous state, presented a severe phenotype. Regarding, the c.3894dupT (Ala1299Cysfs*3) mutation, it was detected in Neuro16 patient, and has been reported in Italian patients [56] and recently in an AT Tunisian patient at a heterozygous state [53]. In addition, the c.7089 + 1G > C variant was predicted to disrupt the splicing donor site leading to exon 48 skipping. This mutation was identified in Neuro6 patient who also harbored the c.5763–2 A > C (Pro1922Asnfs*4) genetic variant. Our previous study predicted that the c.5763–2 A > C mutation induces the loss of the splicing acceptor site and the use of a cryptic splice site 11nt downstream likely leads to a premature stop codon, resulting in a classical AT severe phenotype at homozygous state [52]. The functional effects of identified splicing mutations at mRNA level were assessed and revealed the presence of aberrant transcripts in Neuro6 patient who is compound heterozygous for these mutations, confirming the predicted pathogenic effect of *ATM* genetic variants. Indeed, the presence of aberrant transcript was also confirmed in the parent carrier of one of these mutations. However, it was not possible to assess the profile of the other parent due to biospecimens unaivability. We further examined the expression of total ATM mRNA level. Although we did not assess the expression of each transcript separately, the overall ATM transcript showed a downregulation in Neuro6 patient and his father heterozygous for c.5763–2 A > C, relative to controls. The effect at the expression level was more prominent in patient. Indeed, assessing ATM mRNA expression could reflect the effect of mutation on transcript production or stability, which could subsequently influence the level of functional proteins produced. Many AT mutations cause premature stop codons or splicing defects that trigger mRNA degradation. Indeed, the c.5763–2 A > C has been predicted to induce a premature stop codon, this might elicit the nonsense-mediated mRNA decay (NMD) machinery targeting the aberrant transcript with a premature stop codon for degradation. This could explain the decrease of ATM mRNA level in the parent carrier of this mutation, but still expresses ATM mRNA from the wild-type allele, resulting in only partial decreased ATM transcript level. Conversely, the patient who carriers the same NMD-induced mutation plus the other splicing mutation presented a more markedly overall downregulation of ATM mRNA level, consistent with combined effects on mRNA from both mutations that could affect mRNA stability. Nonetheless, we could not assume whether the pathogenic effect of the c.7089 + 1G > C mutation is reflected only at transcript level or also at protein level. However, our in silico analysis predicted that the c.7089 + 1G > C mutation leads to an inframe deletion probably resulting in the formation of a protein lacking amino acid coded by exon 48–49 (p.Asn2326_Lys2363del). This fragment is part of the tetratricopeptide repeat domain 3 (TRD3) (2195–2476) of FAT domain of ATM protein. The TRD3 subdomain contributes to a large fraction of the ATM dimer interface surface, ensuring extensive contacts with multiple regions between two ATM monomers [57]. Mutation at this site could thereby affects monomer-monomer interactions interfering with ATM dimerization and consequently impairing protein structural integrity. Indeed, the activation of ATM protein requires a transition from homodimer to monomer conformation in response to DNA damages, while an intramolecular disulfide bond is formed between monomers in the dimer structure in response to oxidative stress [58, 59]. Mutations that affect protein dimerization could potentially alter protein activation in response to different stimuli. While the c.7089 + 1G > C (p.Asn2326_Lys2363del38) does not directly affect the kinase domain, but it could potentially influence protein activation and functional regulation.

In addition, our results identified a novel Alu element insertion in *ATM* gene at exon 54 that was predicted to induce a frameshift and a premature termination of the protein translation (p.Asp2661Valfs*7) and consequently it is likely to produce a truncated ATM protein or no detectable ATM protein as a result of nonsense-mediated decay. Due to the unavailability of biological material we couldn’t assess the effect of the mutation at the transcript level. Indeed, the mutational site resides in the kinase domain of the protein, particularly within the N-lobe (2614–2770), resulting in the loss of the C-lobe (2771–2957); a critical subdomain for the catalytic activity of the protein, and downstream elements (PRD and FATC), essential for the kinase activity regulation. Therefore, this mutation could presumably lead to a complete loss of kinase activity, which by definition results in a classical severe AT phenotype. Mutation An insertion of an Alu sequence at intron 54 of *ATM* gene has been previously reported in patients at risk of hereditary breast and/or ovarian cancer (HBOC) syndrom [60]. Despite the contribution of mobile element insertion as disease-causing events, they are generally understudied in rare genetic diseases [61]. A retrotransposon profiling at whole-genome level among AT patients revealed the presence of retroelement insertion in the *ATM* gene, specifically integrated within non-coding regions, which may cause protein loss of function, highlighting their relevance as causative variants and therapeutic targets in AT disease [62].

Furthermore, one missense mutation was identified in Neuro18 patient, notably c.8671G > C (Gly2891Arg), resulting in the substitution of a glycine residue by an arginine. Indeed, this mutation is located in the kinase domain of ATM protein, and missense mutation in this domain could lead to inactive kinase protein “Dead Kinase” [63]. In fact, the c.8671G > C (Gly2891Arg) mutation has not been previously reported in AT disease, however, a c.8672G > A (p.Gly2891Asp) mutation has been described at compound heterozygous state in an AT patient with a mild phenotype, leading to the expression of a mutant ATM protein with a residual kinase activity [64]. In fact, Neuro18 patient presents a late disease onset, manifested at five years old and dominated by extrapyramidal symptoms, which characterize the variant AT phenotype. The c.8671G > C (Gly2891Arg) mutation has been documented in T-cell prolymphocytic leukemia (T-PLL) [43]. Similarly, the patient carrier of the c.8672G > A (p.Gly2891Asp) mutation developed breast cancer and showed a severe clinical radiosensitivity [64]. This highlights the relevance for an early diagnosis of AT disease to provide a timely patient follow-up for a prompt detection of malignancy among carriers of oncogenic ATM variants, including both AT patients and their parents. Missense 3D and Hope tools, have predicted a structural damage induced by this mutation notably due to different physicochemical properties of wild-type and mutant residue. Indeed, this mutation was located within the active site of ATM kinase protein. It is located in the activation loop of the c-lobe of kinase domain (residues 2888–2911), particularly within the DLG motif, which is composed of Asp2889, Leu2890 and Gly2891 residues, known as DFG in other kinase proteins. This highly conserved motif forms a part of ATP-binding site underscoring a critical role in the catalytic activity of kinase protein, which highlights the functional relevance of mutation at this site. Although molecular docking analysis revealed that mutation induced only a marginal reduction of ATP binding affinity, which should be interpreted cautiously the observed changes in interaction patterns upon Gly2891Arg mutation could still suggest a structural effect of the mutation on the ATM-ATP complex, that need further experimental validation. Furthermore, the DFG located at the beginning of the activation loop, undergo conformational changes defining the active and inactive state of the protein, which is crucial for the kinase regulation [65]. The flexibility of the activation loop is conferred from glycine residue. Mutation at this residue introduce steric hindrance which likely interfere with protein flexibility ultimately affecting the activity of the protein. This has been corroborated by CABS-flex analysis revealing a reduced flexibility following Gly2891Arg mutation, which may reduce the activation efficiency of the ATM protein possibly affecting the kinase activity. While CABS-flex has proved computational efficiency for predicting protein structural fluctuations, producing flexibility profiles consistent with experimental NMR data and often performing like short classical molecular dynamic (MD) and being orders of magnitude faster than MD simulation due to its coarse-grained Monte Carlo sampling. Benchmarking studies shown that CABS-flex could even produce distribution of residue fluctuations patterns that align closely with NMR data than MD because of its efficient sampling. Nevertheless, all-atom MD simulations method remains the gold standard for detailed energetic profiling providing more complete representation of protein dynamics, even though at significantly higher computational cost. Combining these approaches, using CABS-flex and MD for an initial screening and a refined atomistic analysis could provide an accurate protein dynamic representation [66, 67]. Taken together, the c.8671G > C (Gly2891Arg) mutation could affect protein structural flexibility which disrupt ATM protein activation interfering with conformational changes required for an optimal protein activation. Furthermore, the effect of the mutation on ATM-ATP complex, may affect the phosphorylation of downstream substrates, given the role of ATP as the phosphate donor, affecting consequently the kinase activity of ATM protein. Functional study is required to confirm these effects. Identification of novel variants in *ATM* gene and exploring their probable mechanisms of pathogenicity could gain deeper insights into the genetic basis of the disease.

Our computational analyses have predicted a pathogenic effect of the novel identified mutations in AT patients, alongside the functional characterization at mRNA level for both splicing mutations which has confirmed the effects of mutations on ATM transcript, nevertheless, it is still mandatory to assess the effect of mutations at protein level; notably through studying the expression of ATM protein and its kinase activity and performing functional validation of the molecular docking and flexibility results to define the effect of mutations on the ATM protein and accurately classify patients carriers of these mutations to classical or variant AT phenotype.

### PBMC: immunological characterization and biomarkers identification

#### Immune profiling

Although AT represents the most frequent Primary Immunodeficiency Disorder (PID) in Tunisia, a comprehensive immunophenotyping of Tunisian patients is yet to be conducted [68]. Furthermore, despite heterozygous carriers of a pathogenic ATM mutation, notably parents and siblings of AT patients, were commonly described as clinically undistinguishable from healthy non carrier individuals, an increased risk of malignancy mainly to breast cancer and cancer of the digestive tract, ischemic heart disease and diabetes have been reported among those carriers associated with a reduced life expectancy [30]. Nonetheless, there is no general consensus on the management of heterozygous carriers of pathogenic ATM mutations worldwide. In this regard, a detailed immunological investigation of AT parents may give insights into immune profile impairment potentially underlying these health risks. Additionally, in an attempt to investigate a possible correlation between genotype and immunological phenotype in AT disease, patients were categorized according to their *ATM* mutational type into two groups. In the present study, our preliminary findings revealed difference of the percentage of different immune cells between AT patients compared to age-matched healthy donors. Regarding LB cells, a trend toward a decreased percentage was noted in AT patients compared to healthy controls and which was slightly more pronounced in AT patients with T/T genotype compared to non-T/T group. Indeed, sinopulmonary bacterial infections have been mainly attributed to B cells deficiency leading to suboptimal B-cell response and antibody deficiency [69], which characterize the classic AT patient group [70]. This may probably explain the difference of LB distribution between the two groups. While no obvious overall difference of the total percentage of LTCD4 + cells was found between the different groups, based on *ATM* genotype, patients with T/T mutation exhibited a reduced percentage of these cells compared to patients with non-T/T mutations, and also relative to controls. Furthermore, our analysis revealed a reduction of LTCD8 + percentage in the majority of AT patients compared to healthy donors, although two out of five patients presenting T/T phenotype displayed a higher percentage of LTCD8+.

Overall, a more reduced percentage of LB, LTCD4 + and LTCD8 + was observed in the majority of AT patients presenting biallelic *ATM* truncating mutations compared to patients with non-truncating mutation which could be associated with a more severe immunodeficiency. In addition, an expansion of senescent T cells in AT patients for both CD4 and CD8 cells with a reciprocal reduction of activated T cells was observed. This is in line with previous report highlighting an aged immune system characterized by reduced naïve T cells accompanied by an increased LT CD27-CD28- which may underly the immunodeficiency observed in AT patients [17]. A reduced number of naïve cells was reported in AT patients, with no changes for memory T cells. This deficiency may account for the reduction of the total LTCD4 + and LTCD8+ [12, 13] and may results from an intrinsic activation defect. Indeed, naïve T cell CD4 + CD45RA + and CD8 + CD45RA+ counts are proportionally much lower than total LTCD4 + and LTCD8 + cell counts due to an increase of differentiated cells [17]. While controversial data exists reporting a similar total LTCD4 + and LTCD8 + cells in AT compared to healthy donors and has been explained by the important expansion of senescent cells at the detriment of activated LTCD27 + CD28+ cells [69]. Our preliminary results demonstrated the same pattern of CD27 + CD28+ and CD27-CD28- for LTCD4 + and LTCD8 + populations in the majority of AT patients from both groups, but which was more prominent in patients with T/T mutations, nonetheless, one patient in each of the group presented different pattern, re-emphasize the interindividual difference of the immunological profile. In addition, it is unclear why, in biallelic null mutations of *ATM* group, patients exhibited an increase of activated LT CD4 + CD27+CD28. While the absence of the co-stimulatory receptors, notably CD27 and CD28 has been widely associated with terminally differentiated and senescent T-cell phenotypes, the immunosenescence process is more complex, as it is associated with broader changes at functional and phenotypic level [71]. Indeed, other changes, notably the loss of CD57 and KLRG1 expression have been explored alongside CD27 and CD28, and has been considered as additional hallmarks of immunosenescence [72]. The absence of data regarding other T cells subsets, such as naïve and T-memory cells in our study could hamper a comprehensive assessment of the widespread immune alterations. This highlights the importance of including these markers for more complete immune profiling for both AT patients and their parents.

Our analysis demonstrated an increase of the percentage of total NK and NKT cells in AT and ATLD1 patients compared to healthy donors. This increase was noted in all affected AT patients independantly of their genotype, however, it seems to be more important in patients with ATM T/T mutations. In fact, LT cells are largely explored in AT disease, nonetheless, data concerning NK cells and monocytes are limited with controversial findings for NK cells. While similar data has been previously documented with an the elevation of the number of NK cells in AT patients that has been explained as a probable compensatory mechanism for T cells immunodeficiency [73], other studies have reported a preserved NK and NKT cell frequency [15]. Further research have pinpointed to the functional defect of NK cells, such as an impairment of NKG2D/NKG2DL pathway [14]. A reduced proportion of CD56dim has been reported concomitant with an unchanged expression pattern of CD57 marker in AT patients [14]. In the present study, AT and ATLD1 patients exhibited an elevation of CD56dim and CD56bright subsets, which may partly reflect the overall expansion of NK cells. A trend towards CD57negNK/CD57pos NK ratio decrease was observed in both patient groups. CD57 data expression was not available for all included patients. CD57 is a well-defined senescent marker for T cells. It is defined as a marker of NK cells’ differentiation and CD57pos NK cells expansion has been described during the aging process. CD57^pos^ NK cells’ accumulation may represent a consequence of cumulative lifetime exposure to infections [74, 75].

Furthermore, our findings described a decrease of the percentage of classical monocytes accompanied with an increase of intermediate monocytes, while non-classical monocyte subsets remained unchanged in AT patients compared to healthy donors. The pattern of classical and non-classical monocytes distribution was noted in the majority of AT patients included in both subgroups, and was more prominent in patients with biallelic truncated *ATM* mutations. Additionally, the majority of patients within this group presented an expansion of non-classical monocytes, which was not clearly evident when assessing the whole AT group as the majority of patients with non-T/T mutations had different profile. Data regarding monocyte characterization in AT disease are scare and have revealed an increase of total monocytes in patients, nevertheless, the distribution of the different subpopulations has not been assessed in the context of AT pathology [15]. Besides, age-associated phenotypic changes in monocytes have been characterized mainly by a significant shift toward an expansion of intermediate (CD14^++^CD16^+^) and non-classical monocyte (CD14^+^CD16^++^) subsets at the expense of classical monocytes (CD14^++^CD16^−^) [76] Monocytes are key mediators of inflammaging process, through their cellular shift that is associated with a functional shift toward a pro-inflammatory phenotype as CD16^+^ positive monocytes are producers of proinflammatory cytokines such as TNF-α and present a reduced function [77]. Inflammaging contributes to immunosenescence, which in turn triggers and exacerbates inflammaging, accelerating the aging process and contributing to age-related diseases [18, 19].

Our preliminary study revealed an increase of LT CD27-CD28- cells which is in line with prior studies, besides an accumulation of total NK cells. Indeed, the accumulation of NK cells accompanied with functional impairment has been reported in relation to the aging process, given the role of these cells as an important actor in the immunosurveillance of senescent cells, their dysfunction contribute to accumulation of senescent cells and subsequently to inflammaging [78]. Premature aging is a significant component in AT disease and represents a crucial driver to disease pathogenesis [21]. The immune system of AT patients has been defined as congenitally aged emphasizing an immune aging [17]. Previous studies highlight that inflammation represent an important factor in AT disease related to different manifestations such as neurodegeneration and autoimmunity, and could contribute to AT pathogenesis, nonetheless, underlying mechanism to chronic inflammation still largely undefined. It has been suggested that sustained DNA damages and oxidative stress related to ATM deficiency could promote cellular senescence which through SASP secretion trigger chronic inflammation [20]. Understanding the interplay between immune dysregulation, notably immunosenescence and chronic inflammation in association with aging hallmarks could decipher molecular and cellular mechanisms behind AT premature aging and that could represent putative targets to alleviate it.

While extensive data concerning immune characterization of AT patients exist, very limited research studies attempted to link the immune profile of AT patients with *ATM* genotype showing heterogeneous results. A study conducted by Staples et al. [26] reported a notable correlation between the genotype and immunological profile, showing that patients with two null mutations associated with no ATM kinase activity present a considerably more severe phenotype at immunological level compared to patients with missense or leaky splice site mutations associated with a residual kinase activity. Patients with no ATM kinase activity presented a lower number of LTCD4+, LTCD8 + and LB cells compared to the other group alongside a more pronounced deficiency of humoral compartment notably of IgA and IgG2 [26]. Other study was carried out based on retrospective data highlighted mainly a difference of LB population, while no obvious difference of humoral compartment or T cells based on *ATM* mutational type, a more severe LB phenotype and a reduced life expectancy was observed in patients carrying biallelic truncating mutations [27].

Despite that our preliminary study was based on a very small number of patients in each group, we tried to perform a deep characterization of immune profile in relation to *ATM* genotype, in an attempt to further assess the genotype-phenotype possible relationship at immunological standpoint in AT disease. Overall, our results outlined that AT patients with biallelic truncated ATM mutations seem to present a more severe immunological phenotype compared to those with at least one non-truncated mutation, and that AT patients with non-biallelic truncated mutations presented for the majority of immune subsets an intermediate phenotype between those with truncated mutations and healthy donors that is more similar to the other AT group. This might be probably related to the residual kinase activity highlighting the important role of ATM protein in immune cells. In addition, we noted an interindividual difference and discordance within the same AT group reflecting a different immune profile. Indeed, difference at clinical and immunological level in siblings having the same ATM mutations has been documented, suggesting the possible involvement of other factors, such as environmental, epigenetic factors or gene modifiers that could influence disease expressivity and phenotypic variability [39, 79]. The characterization of genotype-phenotype correlation could identify genetic defects associated with specific immune profile allows guidance for patient management toward an early and appropriate actions in patient care to prevent health complications, nonetheless, it should be taken into consideration the potential contribution of other factors to the disease expressivity. Further large-scale longitudinal studies should be conducted to better explore this relationship.

While immunodeficiency is a well-reported feature in AT disease, it is rarely observed in ATLD1 and never documented in FRDA diseases [34, 80]. ATLD1 is due to a loss of MRE11 protein expression which is mainly involved in DNA repair. As for AT, ATLD1 phenotype belongs to the group of progeroid syndromes induced by mutations in genes involved in DNA-repair pathways [81]. The decrease of activated T cells we observed in our study may be associated with the senescent phenotype. In addition, while earliest studies have not reported a cancer proneness as a feature in ATLD1 unlike AT patients, some ATLD1 patients developed cancers [82]. The effect of MRE11 mutation on the MRE11/RAD50/NBS1 (MRN) complex structure has been suggested to explain in part the difference of cancer predisposition among ATLD1 patients [83]. T cell co-stimulatory receptors play an important role in anti-tumor immune response, associated with the role of T cells in the clearance of precancerous cells and the immunosurveillance of cancer [84]. Though we cannot assume that the downregulation of CD27 and CD28 expression observed in our patients may contribute in part to the susceptibility to cancer development observed in some ATLD1 patients, a longitudinal study of immune profile of ATLD1 patients associated with cancer development is warranted. While we were limited by the number of samples given the rarity of this disease in Tunisia and worldwide, our findings point to the possibility of a broad trend toward alteration in specific immune subsets in ATLD1 patients that might be related to the role of disease causative gene in DNA damage repair and which may be involved in the disease pathogenesis. Future studies are needed to evaluate immune aspect in this group of rare disease.

Regarding FRDA, it is due to mutation in the Frataxin gene coding for a mitochondrial protein Frataxin. while transcriptomic and proteomic profiling of PBMC in FRDA disease have been conducted to identify peripheral molecular signatures associated with disease pathology highlighting the presence of inflammatory signature [85, 86]. Our results have demonstrated a similarity of the immune profile of FRDA patients compared to healthy donors, however a heterogeneity of the percentage of CD56dim NK cells was observed between the siblings.

In our study, the immune profile of parents relatively mimics their children, presenting similar but milder impairments of T-cell compartment and a cellular shift of monocyte subsets. This cellular shift could reflect an inflammaging state that may contribute to the higher incidence of age-related diseases observed in those carriers of ATM mutations, which underscore the necessity for further exploration. While monocyte subsets distribution has not been previously assessed in AT parents, findings concerning the other cell types were in accordance with a previous study. This study revealed a reduced proportion of naïve LTCD4 cells and recent thymic emigrant (RTE) cells conversely to an elevated effector memory-like LTCD4 in both patients and parents compared to healthy donors [33]. The observed cellular defect mimicking AT patients has been related to recurrent infections observed in some patients’ parents which may be linked to defective DNA repair [33]. In our study, we aimed to characterize the immune profile of AT parents by exploring co-stimulatory receptors expression. An expansion of senescent LTCD8 similar to affected patients was recorded, which may contribute to the high risk of infections and malignancies observed in proband’s parents, as LTCD8 play a crucial role in the clearance of infected or transformed cells. Interestingly, an elevated proportion of LTCD4 CD28- cells was observed among parents. Indeed, an increased DNA double-strand breaks (DSB) lead to high DNA damage in AT parents T cells has been related to lymphocytes subsets alteration, notably among parents with recurrent infections [33]. The role of DNA damage in driving T cells senescent phenotype is reported in an ATM-dependent manner [87]. CD28 expression is lost following repeated antigen stimulation and with aging [88]. Indeed, while LTCD4 cells are more resistant to age-related changes than LTCD8 cells, a progressive elevation in the percentage of LTCD4 cells lacking CD28 marker expression correlated with a decline of its function is common with increasing age in healthy subjects and in patients with chronic infections [89]. The accumulation of CD28null T cells correlates with the development and the progression of various diseases, and their potential as putative immunological biomarkers and targets to delay the aging process and mitigate age-related diseases [90]. In fact, LTCD4 CD28null cells accumulation contributes to the pathophysiology of acute coronary syndromes [91] and has been reported to be associated to the occurrence of first cardiovascular event and to a poor prognosis after an acute coronary syndrome among patients with diabetes [92]. This may potentially contribute in part to the high risk of cardiovascular diseases observed in AT parents. Further research elucidating the role of LTCD4 CD28^null^ CD27 + in the immune response and their contribution to age-related diseases remains of importance. Our preliminary findings describe a potential similarity of the immune profile between AT parents and their children, nonetheless, we cannot exclude the possible effects of confounding factors, notably age, environmental or epigenetic factors, although we tried to minimize these factors by comparing the immune profile of AT parents with an age-matched healthy donors group to reduce the potential influence of age on immune profiling. Our preliminary findings identify immune alterations potentially associated with an immune aging phenotype in AT parents that could be related to ATM deficiency associated with ATM heterozygosity. This may be supported by previous evidence of the involvement of DNA damages directly linked to ATM loss in cellular immune alterations observed in AT parents [93], and the key influence of ATM deficiency in driving the aging process [21]. In view of the exploratory nature of our study, results should be interpreted with caution and underscore the need for further exploration of immune aging phenotype in AT parents and among carriers of *ATM* heterozygote mutations which may contribute to age-related diseases permitting to identify immune signature that permit to detect patients at risk of these morbidities.

#### Gene expression analysis

Studies based on transcriptomic profiling of PBMC from AT patients are limited [23, 39, 40]. RNAseq analysis followed by clustering revealed differences in the gene expression profiles defining multiple phenotypic subtypes [40]. Our analysis match these previous observations revealing an heterogenous expression pattern among AT patients. Evidence from different AT models, based on transcriptomic profiling, notably from patients post-mortem tissues, mice models and cellular models have revealed a notable widspread changes of gene expression profiling at peripheral level and central nervous system level as a result of ATM activity loss. This has been explained in part by chromatin accessibility and RNA-loops formation in a ROS dependant manner that correlate with GC content [94], and that difference of transcriptional profiling could underpin phenotypic variability in AT patients [39]. However, the involvement of gene expression regulators has not been widely characterized. As AT disease is based on their possible link to AT pathogenesis, given their well defined role in stress response signaling and immune regulation. In fact, AT cells are characterized by chronic cellular stress due to DNA damages and oxidative stress induced by ATM loss that has been demonstrated to elicit a chronic activation of stress response signaling [95].

Indeed, our PBMC-based gene expression analysis revealed an upregulation of *FOXM1* gene in the majority of analyzed patients. FOXM1 is a transcription factor controling cell cycle progression and genome stability. It is an oncogenic factor, which has been correlated to the onset and progression of cancer [96]. *FOXM1* overexpression has been reported to be related to many types of cancer including leukemia, notably the B acute lymphoblastic leukemia, which is the most frequent cancer documented in AT patients [97, 98]. Its dysregulation may therefore contribute to the higher risk of malignancy observed in AT patients. Furthermore, an altered expression of *FOXO3*, *METTL3* and *IL33* genes was detected in Neuro8 and Neuro10 patients, both are homozygous for truncated *ATM* mutations. At clinical level, Neuro8 patient showed a more severe neurological phenotype compared to other patients included in our cohort. On the other hand, the age of Neuro10 patient suggests a more advanced stage of the disease. Additionally, Neuro10 presents a neurogenic symptom characterized by distal amyotrophy at an early stage of the disease, manifested at 12 years old. As AT is a progressive neurodegenerative disease which is manifested by progressive neurological decline, the disease severity could be possibly in part estimated as the disease duration in the absence of a score that could accurately assess the severity of the phenotype. In fact, diffuse cerebellar degeneration involving both hemispheres and vermis is a hallmark feature of AT disease. Histopathological evidence of pontocerebellar pathways atrophy and demyelination of corticospinal tracts in AT has also been revealed. In general, theses neuropathological impairments were based on post-mortem autopsies, usually at the end stage of the disease. These alterations are usually described in old AT patients and rarely in young patients [99]. Previously published data documented that with increasing age, neuropathological changes become more advanced and severe, but there is a substantial individual variation. Thus, we suggest that *FOXO3*, *METTL3* and *IL33* may be related to disease severity or progression in these patients. These dysregulated genes have potential roles in both immune regulation and neurodegeneration.

In fact, FOXO3 is a key transcription factor that behaves as a sensor regulator activated upon cellular stress and controls different processes by regulating genes involved in cellular senescence, apoptosis, proliferation, cell cycle arrest, antioxydant system, DNA repair, acting as a central mediator of cellular stress response [100, 101]. In addition to its nuclear and mitochondrial functions orchestrating the fine-tuning transcriptional programs in both compartments, FOXO3 mediates the nuclear-mitochondria crosstalk, hightening the complexity of the intricate stress response network restoring cellular homeostasis [102]. Based on experimental cellular model, a direct functional interaction between FOXO3 and ATM protein has been reported in the DNA damage response pathway [103] FOXO3 plays an important role as a regulator of *ATM* expression in response to oxidative stress [110]. This highlights the relevance of this protein in ATM signaling [104].

Moreover, according to previous RNAseq dataset, an overexpression of FOXO3 has been recorded in PBMC from AT patients compared to healthy controls [23]. FOXO3 overexpression might indicates an attempt to counteract cellular stress but its excessive expression could promote cellular apoptosis and neuronal cell death contributing to the progeression of neurodegenerative diseases, such as Alzheimer disease (AD) [105] and Parkinson disease (PD) [106]. FOXO3 also regulates immune cell function modulating key signaling pathways [107]. Indeed, besides DNA damages AT cells are characterized by an increased oxidative stress [108], which elicit a cellular stress response. All these evidence suggest a possible contribution of *FOXO3* in AT disease. Our preliminary findings showed an upregulation of *FOXO3* expression in two AT patients which could represent a possible response to counteract cellular stress caused by ATM loss which may be more prominent in these patients. The upregulation of *FOXO3* could merely be involved as part of cellular stress response or could also contribute in turn to the severe neuropathological impairment enhancing neuronal cell death and immune alterations.

Several studies point to the regulation of FOXO3 activity that is mainly attributed to post-transcriptional modifications which dictate its cellular localisation, nonetheless, the contribution of epitranscriptomic modifications in *FOXO3* gene expression regulation has only recently been investigated [109, 110].

Indeed, epitranscriptomics or “RNA epigenetics” refers to post-transcriptional mRNA reversible modifications, which are dynamically controlled in response to different stimuli and cellular stress modulating gene expression. The m^6^A represents the most frequent mRNA modification and METTL3 a m^6^A methyltransferase, is the core catalyst of m^6^A methylation [111]. Among its targets, METTL3 protein regulates ATM and downstream signaling in the DNA damage response through m^6^A modification, and in turn, it is an ATM target activated by ATM-mediated phoshorylation in response to DNA damages involved in homologous recombination (HR)-mediated double strand break repair (DSBR) [112, 113]. Increasing evidences support the contribution of epitranscriptomic modifications, notably m^6^A in various neurodegenerative diseases [114]. Aberrant m6A methylation and *METTL3* upregulation have been reported to be associated with Tau protein accumulation in brain postmortem tissues from AD patients and mice models [115, 116]. An elevated RNA m^6^A level has been described in glial cells in the hippocampus and cortex of AD patients, whereas a *METTL3* reduction-mediated m^6^A dysregulation has been documented in pyramidal neurons of the hippcampus resulting in an elevated oxidative stress and a disrupted cell cycle regulation ultimately leading to a notable neuronal cell death [117]. Despite these discrepancies regarding m^6^A levels in AD, existing data support the potential contribution of dysregulated epitranscriptomic mechanisms to AD pathogenesis. Additionally, the alterations of m6A profiling and m^6^A -related proteins expression levels have been described at PBMC level in AD [118] and PD [119] patients suggesting their potential as putative peripheral biomarkers. In fact, transcriptome-wide methylation profiling of different brain regions has shown that the cerebellum represents the hightest m^6^A methylation levels [120]. Reports have documented the crucial role of METTL3-mediated m^6^A modification in the normal development and physiological function of the cerebellum [121, 122]. In the other side, purkinje cells-specific conditional Wtap knockout mice model has been proven to induce an early-onset cerebellar ataxia associated with an extensive neuronal degeneration through disrupting METTL3/4 expression and activity and thereby m^6^A modification mechanism, emphasizing the significance of epitranscriptomic modifications in cerebellar neuronal degeneration [123]. Furthermore, METTL3 is an important regulator of various immune and inflammatory processes ranging from homeostasis maintenance to functions regulation [124]. In fact, the m^6^A methylation changes and their underlying pathological mechanisms have not been previously explored in the context of AT disease. Our study reported a preliminary finding regarding an upregulation of *METTL3* in two AT patients with clinical severe phenotype. Given the central role of this gene in epitranscriptomic regulation, one plausible explanation is that this change of METT3 might be associated with an overall or gene-specific differential m^6^A profiling contributing in part to differentiel gene expression that could be associated with the severe phenotype observed in these patients, however, further work is needed to confirm this.

IL33 is a newly identified member of IL1 cytokine family and is a pleiotropic cytokine. While it was traditionnally known to function through an extracellular pathway via ST2 signaling serving as an “alarmin” in response to cellular stress and modulating inflammtory and immune responses, a nuclear transcriptional regulatory role has also been assigned [125]. Indeed, ST2 receptor is expressed on the majority of hematopoetic cells highlighting its role in immune-related diseases [126]. Among other cells, ST2 is also expressed on microglia and astrocytes [127]. It is a crucial regulator of immune response and of central nervous system homeostasis [128]. Furthermore, an impaired IL33/ST2 signaling has been reported in several neurodegenrative diseases, where it could represent a dual function as a neuroprotector or a neurotoxic factor [129]. It has been repoted that the decreased IL33/ST2 signaling in the CNS could result in a chronic neuroinflammation in AD and that IL33 restoration could alleviate AD-related neuropathology [130], while an increased level in the periphery and in the CNS has been documented in Multiple sclerosis (MS) patients pointing out its involvement in MS pathogenesis and suggesting a possible role of this cytokine in immune response both at the periphery and within the CNS [131]. IL-33 presents pro-inflammatory as well as anti-inflammatory properties depending on the context [132].

Conversely, there is no existing evidences of *IL33* direct interaction with ATM signaling or involvement in AT pathophysiology, as the expression and the functional role of this cytokine in AT disease or in other forms of ataxia have not been explored yet. Nonetheless, an elevated peripheral cytokine levels, notably of IL6 and IL8 have been associated to comorbidities in AT patients [133]. In addition, other IL1β family members, notably IL1 has been significantly incriminated in the cerebellar neuropathology of AT disease linked to its pro-inflammatory role [134]. Our results described an upregulation of IL33 gene in Neuro8 and Neuro10 patients, while less prominent this overexpression has also been noted in Neuro6 and Neuro18 patients, pointing out to the possible role of this cytokine in AT pathology. In fact, NF-kB has been reported as a transcriptional regulator of *IL33* gene, in turn among its function IL33/ST2 activates NF-kB signaling. NF-kB inflammatory response has been previously described as an enriched signaling by transcriptional profiling of PBMC from AT patients [23]. The observed dysregulation of IL33 expression in PBMC in our cohort, could simply reflect an altered peripheral inflammatory or immune profile which characterize the disease, however, the more elevated level specifically in patients with clinically more severe neurological phenotype might suggest a link between peripheral IL33 level and the neurological phenoype. Intriguingly, previous studies have demonstrated a potential correlation between the peripheral inflammatory phenotype assessed by cell counts, and the severity of cerebellar ataxia in spinocerebellar ataxia (SCA) patients [135, 136]. While remains speculative, owing to the dual role of IL33 in immune regulation, the obseved IL33 elevation could represent a systemic response reflecting a more activated immune response associated with the severe neuropathological phenotype or a compensatory mechanism aiming to mitigate neuroinflammation. This raises the need to further explore the expression of *IL33* and its downstream signaling both in the periphery and the CNS and clarify its role in AT pathology. In addition, while earlier research efforts have tried to characterize the peripheral immune profile of AT patients aiming to better undrestand the effect of ATM deficiency on immune cells and to correlate these alterations with comorbidities, no study has attempted to link peripheral immune alterations to the neurological phenotype in AT disease which could identify novel role of immune regulation in this disease.

The dysregulation of these genes may predict a dysfunctional interplay between RNA regulation, oxidative stress response, and inflammation, which could be related to disease severity/ progression in AT patients. As transcriptional program is associated with AT phenotypic variability, we could not assign if the dysregulation of these genes arises as a cause or a consequence of a more severe course. This is the first report investigating these genes in the context of AT disease. Further research are therefore mandatory to explore their relevance in AT pathogenesis based on larger cohort and longitudinal analysis to assess their expression level changes during the disease progression.

The limitations of our study are primarily attributed to the small sample size of our cohort due to the rarity of these diseases, that reflects the preliminary nature of our findings notably for immune profiling and gene expression analysis. In addition, while we tried to assess gene expression profile related to the disease severity based on the clinical phenotype and the disease duration, the unavailability of an homogenous score that permits to accurately assess the severity of the neurological phenotype in our AT patients’cohort represents another limitation. Furthermore, functional validation of novel variants pathogenecity using western blot aiming to assess ATM protein expression and its kinase activity are mandatory to clearly classify patients into classical and variant groups, which was absent in this study. In addition, the lack of longitudinal data represents an other significant limitation to assess the potential of identified immune alterations and gene expression changes as relevant biomarkers. Due to the small number size and the absence of longitudinal analysis we have conducted a descriptive analysis rather than causal associations. Nevertheless, despite these limitations, our preliminary study further characterize the immune abnormalities in AT patients and identify common signature in AT patients and parents. Furthermore, we provide some important candidate genes that deserve further investigation in the search of their utility as peripheral biomarkers associated with disease severity or monitoring progression, and which may provide novel insights into AT pathogenesis. Such biomarkers may then offer a target to prevent or slow the disease progression. Future research with larger cohort, based on longitudinal analysis are needed to confirm our results wich could provide a more granular perspective into the immune aging and the role of these genes in AT.

In summary, our results identify novel *ATM* mutations in AT disease and demonstrates the relevance of the exploration of the putative mechanisms of variant pathogenecity. Furthermore, our study provides a comprehensive charaterization of immune profile identifying immune differences in AT patients associated to *ATM* mutational type and report immune similarities between AT patients and their parents. The present study further identify gene expression changes that could be linked to AT disease severity.

## Conclusion

Our findings extend the mutational landscape of AT patients worldwide and highlight the involvement of mobile element insertion as causative variants in this disease. We identified and characterized the functional effect of two splice site mutations on ATM transcript. Structural bioinformatic analysis suggests an impact of identified missense mutation on ATM-ATP complex stability, and also altering the protein flexibility. Furthermore, immune profile changes highlight a senescent phenotype which is more prominent in LTCD8 among both AT patients and their parents and an increased rate of intermediate and non-classical monocytes accompanied with a decrease of classical monocytes in AT patients with biallelic truncated mutations and AT parents. Nonetheless, a mild or no impairment of peripheral immune profiles was observed in other forms of autosomal recessive cerebellar ataxia. Besides cellular impairment, our preliminary study delineated molecular alterations in PBMC based on gene expression analysis, identifying putative candidate genes that may be relevant in AT disease.

## Supplementary Information

Below is the link to the electronic supplementary material.

Supplementary Material 1

## Data Availability

All data generated or analyzed during this study are included in this published article and its Additional file 1.

## Abbreviations

AT: Ataxia telangiectasia
ARCA: Autosomal recessive cerebellar ataxia
DDR: DNA Damage Response
CSR: Class switch recombination
ATLD1: Ataxia telangiectasia-like disorder1
FA: Friedreich ataxia
PBMC: Peripheral blood mononuclear cells
ACMG/AMP: American College of Medical Genetics and Genomics and Association for Molecular Pathology
AFP: Alpha-foetoprotein
MRI: Magnetic Resonance Imaging
T-PLL: T-cell prolymphocytic leukemia
RMSF: Root Mean Square Fluctuation
HBOC: Hereditary breast and/or ovarian cancer
PID: Primary Immunodeficiency Disorder
AD: Alzheimer disease
PD: Parkinson disease
HR: Homologous recombination
DSBR: Double strand break repair
