# Supplementary material for: Novel genetic variants identification and immune profiling in ataxia telangiectasia patients
Source: J Transl Med. 2026 Feb 19;24:422. doi: 10.1186/s12967-026-07871-2 (PMC13020073; doi:10.1186/s12967-026-07871-2)
Supplement: Supplementary file 1 — Supplementary Material 1 [file 12967_2026_7871_MOESM1_ESM.docx]

## Supplemental Figures

**
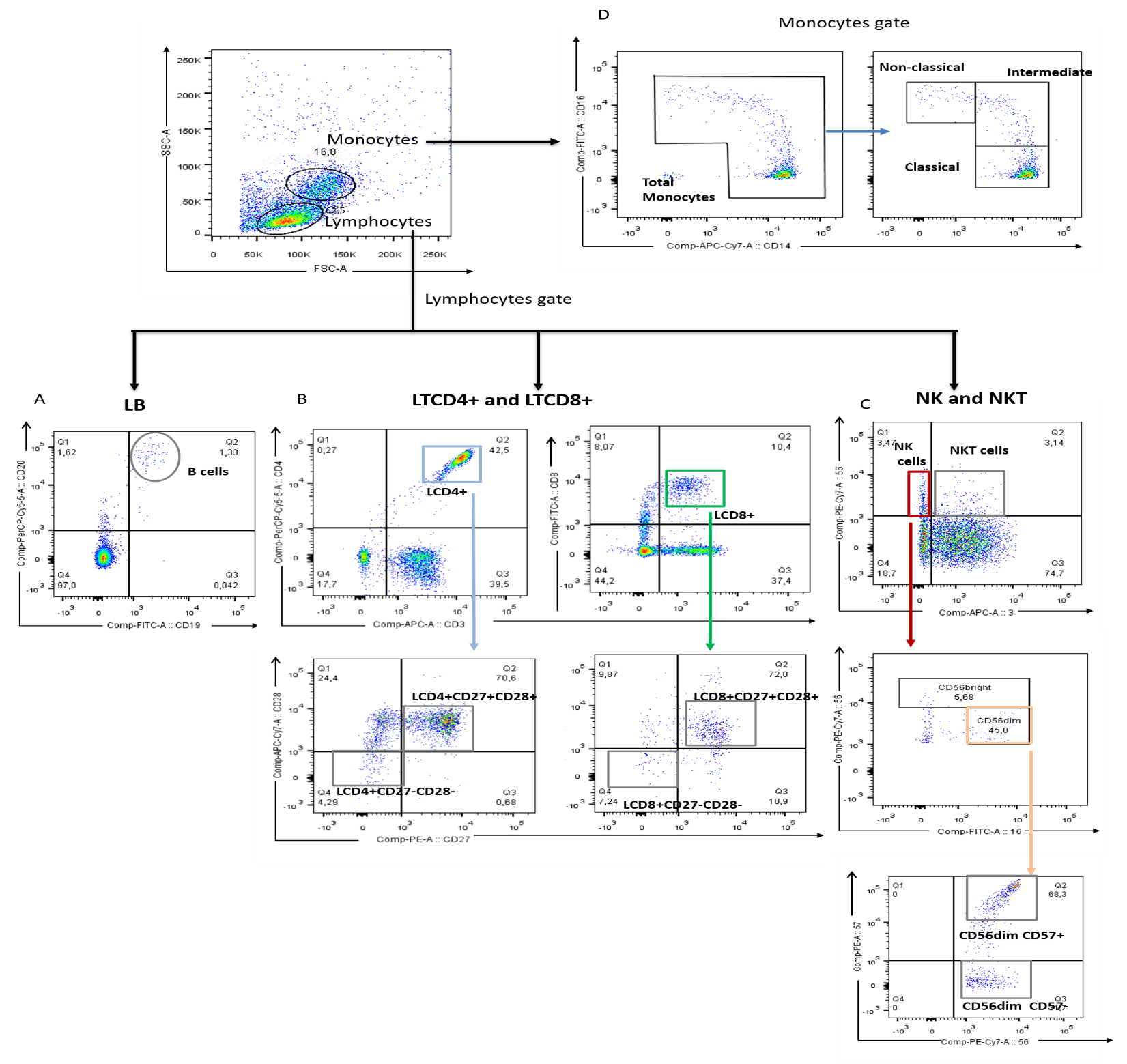
**

**Figure S1: Gating strategy for PBMC subpopulation based on the expression of different surface markers**

1. Lymphocytes and monocytes were identified and gatted based on the forward and side scatter. (A) B cells were defined as positive for CD19 and CD20. (B) T cells subsets were defined from total lymphocytes based on their CD4 or CD8 expression. Cells were then analysed for CD27 and CD28 expression. (C) NK cells were defined as CD3-CD56 + cells, NKT cells were defined as CD3+CD56+. NK cells were further assessed according to the intensity of the CD56 and CD16 expression. NK CD56dim were divided based on CD57 expression. (D) Monocytes were divided based on CD14 and CD16 markers expression; classical (CD14^++^CD16^−^), intermediate (CD14^++^CD16^+^) and non-classical (CD14^+^CD16^++^) monocytes.

**
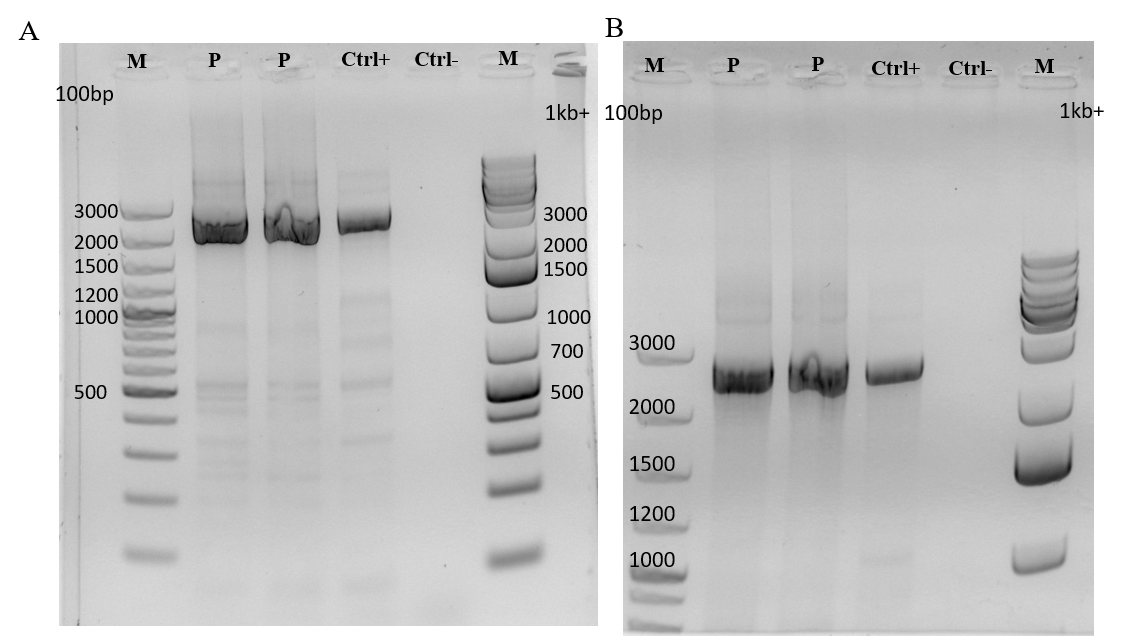
**

**Figure S2: Agarose gel electrophoresis of PCR amplicon after long range PCR**

Long range sequencing followed by migration for (A) 40 min and (B) 1h30 min showing negative result for Alu sequence amplification. Lane M indicates the DNA marker, P: patient, Ctrl+: positive control, Ctrl-: negative control.


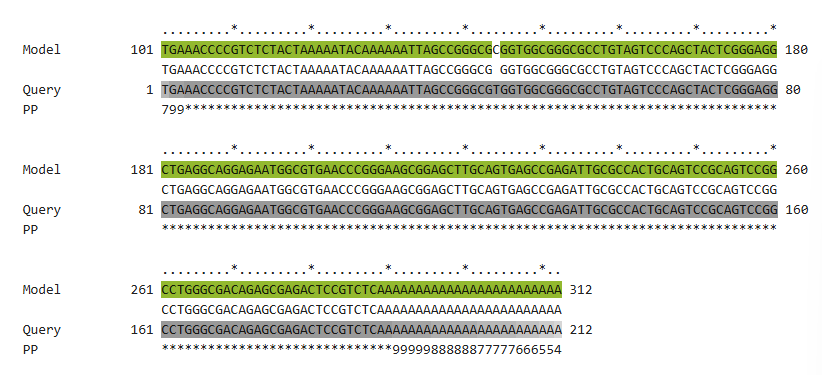


**Figure S3: Sequence alignment with AluYb8 using Dfam**

The model line corresponds to the consensus sequence for AluY aligned state in the model, colored according to match with query sequence. The query line presents the input sequence. The PP line represents the degree of confidence in each aligned residue. *: meaning highest confidence.


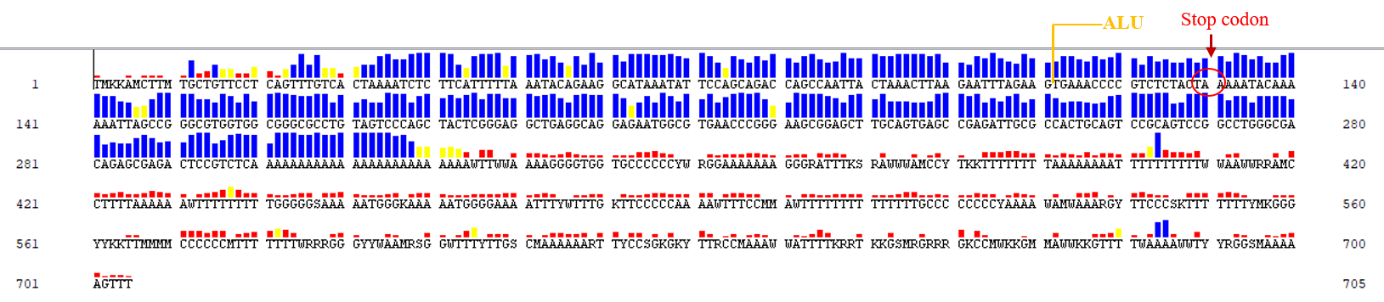


**Figure S4: Predicted consequence of Alu element insertion in exon 54**

Coding sequence of exon 54 of *ATM* gene following Alu element insertion demonstrates the creation of a premature stop codon. Alu insertion site in the coding sequence is indicated in yellow, the stop codon is encircled in red.


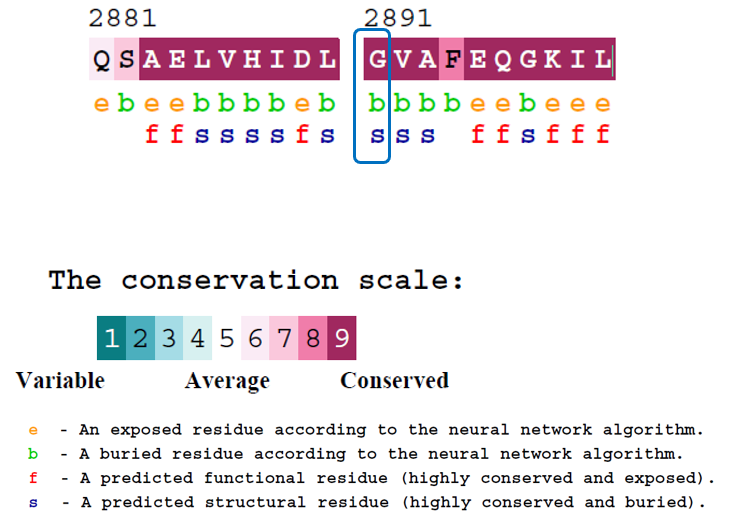


**Figure S5: Conservation analysis of the ATM protein sequence at c.8671G position using ConSurf tool**

The residue at position 2891 of the ATM protein is highly conserved presenting a score of 9. Blue rectangle indicates the position of the mutated residue. b:buried residue, s: structural residue.


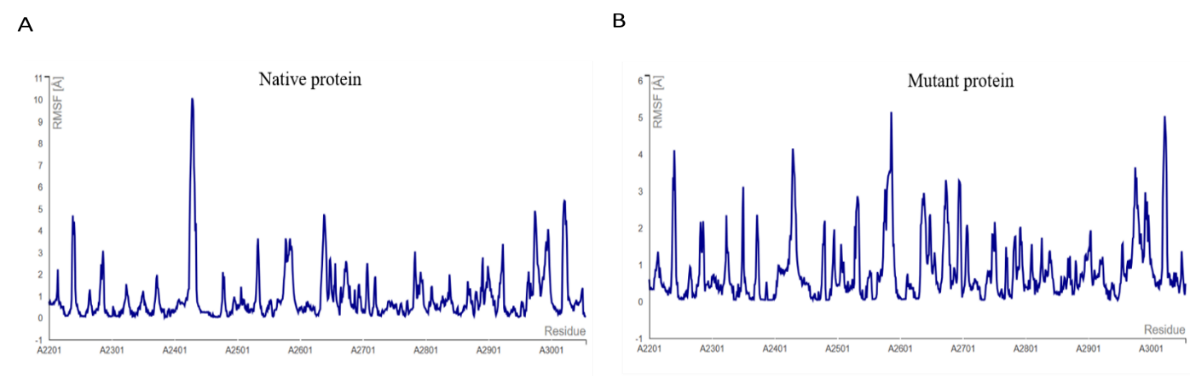


**Figure S6: Graphs depicting the RMSF calculated by CABS-flex 2.0**

Comparison of flexibility profiles between (A)WT and (B) mutant upper protein structure following Gly2890 mutation. RMSF indicates the average positional fluctuation of each residue during the simulation.

**2 Supplemental data**

**Retroelement**

Genomic location: chr11: (hg38)

Orientation: sense relative to the *ATM* gene

Subfamily: AluYb8

Length: 213pb

Target site duplication sequence (TSD): 5’-AAGAATTTAGAAG-3’

TSD length: 13pb

**Sequence and annotation**

• Black: *ATM* exon 54

• **Bold Black and Underline**: integrated retroelement

• *Italic:* poly-A tail sequence. (The length of poly-A tail is hard to precisely determined based on sequencing results)

• **Bold blue**: target site duplication sequence.

• Red: internal mutations within the retroelement.

◼ 5’ truncation from position 1 to 100*

◼ C to T mutation at position 144*

* Positions relative to AluYb8 dfam consensus.

AAGGCATAAATATTCCAGCAGACCAGCCAATTACTAAACTTAAGAATTTAGAAG**TGAAACCCCGTCTCTACTAAAAATACAAAAAATTAGCCGGGCGTGGTGGCGGG**C**GCCTGTAGTCCCAGCTACTCGGGAGGCTGAGGCAGGAGAATGGCGTGAACCCGGGAAGCGGAGCTTGCAGTGAGCCGAGATTGCGCCACTGCAGTCCGCAGTCCGGCCTGGGCGACAGAGCGAGACTCCGTCTC***AAAAAAAAAAAAAAAAAAAAAAAAA*AAGAATTTAGAAGATGTTGTTGTCCCTACTATGGAAATTAAG

## Supplemental Tables

| **Parameters/ Patient ID** | | **Neuro2** | **Neuro6** | **Neuro8** | **Neuro15** | **Neuro16** | **Neuro18** |
| --- | --- | --- | --- | --- | --- | --- | --- |
| **Sex** | | M | M | F | F | M | M |
| **Geographical origin** | | Sidi Bouzid | Kairouan | Kasserine | Kasserine | Tunis | Sidi Bouzid |
| **Consanguinity** | | + | + | + | + | - | + |
| **Age (years)** | | 14 | 14 | 13 | 17 | 12 | 10 |
| **Age at diagnosis (years)** | | 10 | 7 | 6 | 6 | 9 | 7 |
| **Age at disease-onset (months)** | | 17 | 36 | 24 | 13 | 14 | 60 |
| **First revealing symptoms** | | Gait Instability | Gait Instability | Gait instability | Gait instability | Gait instability | Articulation difficulties and tremor |
| **Neurological findings** | **Cerebellar Syndrome**  - Walking capacity  - Cerebellar Ataxia  - Dysmetria  - Hypotonia  - Adiadochokinesis  - Dysarthria  - Tremor  - Nystagmus | Lost  +  +  +  +  +  -  - | +  +  +  -  +  +  -  - | +  +  +  +  +  +  +  + | Lost  +  +  -  +  +  +  + | +  +  +  -  -  +  +  - | +  +  +  -  +  +  +  + |
|  | **Pyramidal Syndrome** | - | - | + | - | - | - |
|  | **Extrapyramidal Syndrome**  - Parkinsonism  - Dystonia  - Chorea  - Myoclonus | -  -  +  - | -  +  -  - | -  -  +  - | -  -  +  - | -  -  -  + | -  +  +  + |

**Table S1: Clinical characteristics of AT patients**

| **Ophthalmolgical features** | Ocular Telangiectasia | + | + | + | + | + | + | + |
| --- | --- | --- | --- | --- | --- | --- | --- | --- |
|  | Oculomotor Apraxia | + | + | + | - | + | + | + |
| **Recurrent infections** | | + | - | + | + | + | - | + |
| **Clinical immunodeficiency** | | + | - | + | + | + | - | + |
| **Cancer (History of cancer development)** | | - | - | - | - | - | - | - |
| **Dermatological features** | Cutaneous telangiectasia | - | - | - | - | + | - | - |
|  | Vitiligo | - | - | - | + | - | - | - |
|  | Others | - | - | - | + | - | - | café-au-lait spots, hypo-pigmented macules and vitiligo |
| **Imaging findings** | Cerebellar atrophy | + | + | + |  | - | - | + |
|  | Peripheral neuropathy | - | + | - |  | - | - | - |
| **Laboratory findings** | AFP (ng/ml) | 289.2 | NA | 44.07 | 38.16 | 46 | 137 | 24.11 |
|  | Ig (g/l)  IgA  IgG | 0.785  NA | NA  NA | 0.01  NA | 0.14  ND | 0.17  NP | NP | 0.29 |
|  | Lymphopenia | **-** | **-** | **-** | **-** | **+** | **-** | NA |
| **Cytogenetic analysis** | | NP | 46,XY | T(7,14)(p14,q11) | NP | NP | NP | NP |

NP: Not Performed NA: Not available

**Table S2: PCR primers used for Alu sequence amplification**

| **Primers** | **Primer Sequences (5’-3’)** | **Exon** |
| --- | --- | --- |
| ATM_53F  ATM_55R | 5' TTC-CAT-TGT-CTA-GAT-TTG-TGC-AT 3'  5' AAA-TGG-AGA-AAA-GCC-TGG-TTC 3' | 53-55 |
| ATM_54seqF1  ATM_int54-seqR1 | 5' TGG-TCT-AGT-TAC-CCT-TGT-CAG-G 3'  5' CTT-TAG-GCC-GGG-TGT-GGT-GG 3' | 54 |
| ATM_54seqF2  ATM_int54-seqR2 | 5' GGG-GCC-AGT-GGT-ATC-TGC-TG 3'  5' CAT-TTT-CTA-AAC-TTA-TAA-CCC 3' | 54 |

**Table S3: Identified variants and their classification according to the ACMG/AMP guidelines**

| **Mutation** | **Genomic position** | **Protein change** | **ACMG criteria applied** | **Interpretation** |
| --- | --- | --- | --- | --- |
| c.5763-2A>C | chr11-108310158- | p.? | PVS1, PM2, PP3, PP5 | pathogenic |
| c.7089+1G>C | chr11-108327759 | p.? | PVS1, PM2, PM4, PM3, PP3 | pathogenic |
| c.2135C>G | chr11-108256225 | Ser712* | PVS1,PM2,PP5 | pathogenic |
| c.3894dup | chr11-108284371 | Ala1299Cysfs*3 | PVS1 PS3,PM2,PP5 | pathogenic |
| c.7981_7982insAluYb8 | Chr11-108333939_108333940 | p.? | PVS1,PM2 | likely pathogenic |
| c.8671G>C | chr11-108347365 | Gly2891Arg | PM2, PM5, PP3, BP1 | likely pathogenic |

PVS: Very strong evidence of pathogenecity; PV: Strong evidence of pathogenicity; PM: Moderate evidence of pathogenicity;PP: Supporting evidence of pathogenicity
